# Do virtual reality-based therapies affect symptomatology and psychosocial functioning in schizophrenia spectrum disorders: systematic review and meta-analysis

**DOI:** 10.1192/bjo.2026.12012

**Published:** 2026-06-18

**Authors:** George Alexander Colgan, Kelvin Kwok Lap Ng, Dan Siskind, Mike Trott, Urska Arnautovska

**Affiliations:** Faculty of Medicine and Biomedical Sciences, The University of Queensland, Australia; Metro South Hospital and Health Service, Brisbane, Australia; Princess Alexandra Hospital Southside Clinical Unit, Faculty of Medicine, The University of Queensland, Australia; School of Medicine, University of Queensland, Australia; Addiction and Mental Health Services, Metro South Health, Brisbane, Australia; Queensland Centre for Mental Health Research, University of Queensland, Australia; Queensland Centre for Mental Health Research, Faculty of Medicine, The University of Queensland, Australia; Faculty of Medicine, The University of Queensland, Australia

**Keywords:** Evidence-based mental health, meta-analysis, psychological treatments, psychotic disorders/schizophrenia, medical technology

## Abstract

**Background:**

Virtual reality is a promising intervention for schizophrenia spectrum disorders (SSDs), offering immersive environments to support therapy. However, evidence for its effectiveness across symptom domains remains inconsistent.

**Aims:**

To assess the effectiveness of virtual reality-based therapies (VRT) for SSDs on symptomatology and psychosocial functioning compared with control conditions.

**Method:**

A systematic review and meta-analysis of controlled trials using virtual reality for individuals with SSDs was conducted. Searches were conducted across PubMed, PsychINFO, EMBASE, CINAHL and Cochrane Central Register of Controlled Trials, from inception to March 2025. Random-effects models estimated pooled effect sizes (Hedges’ *g*) across outcomes, including Positive and Negative Symptom Scale, depression, paranoia and cognition. Risk of bias was assessed using the Joanna Briggs Institute Risk of Bias Tool.

**Results:**

From 2878 unique studies, 9 trials were eligible for meta-analysis. VRT was significantly more effective than control conditions in reducing overall psychotic symptoms (Hedges’ *g* = 0.53, *p* = 0.037). No significant effects were found for other domains (positive, negative, depressive or paranoia symptoms).

**Conclusions:**

Virtual reality therapies are moderately effective at reducing overall psychotic symptoms. Unlike prior reviews, this study did not find significant effects on positive symptoms, possibly due to the heterogeneity of outcomes reported in existing interventions.

**Trial registration:**

PROSPERO: CRD42023470849

Schizophrenia, affecting 1% of the global population,^1^ is a chronic and severe mental illness (SMI) associated with some of the highest disability burden among medical illnesses.^2–4^ Core symptoms include persistent distressing hallucinations, as well as negative symptoms of avolition, impaired social skills and cognitive impairment.^5,6^ Together, these symptoms can severely impact psychosocial functioning and cause feelings of anxiety, depression and suicidality.^7–9^ Although antipsychotics are the first-line treatment for schizophrenia, navigating undesirable side-effects is a challenge to the therapeutic alliance and can impact adherence. Non-pharmacological interventions, with the most prominent of these being cognitive–behavioural therapy (CBT), have been shown to improve both overall symptom burden and functional capacity.^10^ However, a variety of factors, including challenges with motivation, social anxiety and cognitive function, hamper the efficacy of these interventions.^11^ Although traditional therapies initially showed promising evidence for their efficacy, more guarded evaluations – particularly regarding their role in treating negative symptoms – have been raised.^11–13^ One criticism of historical approaches is their ecological validity: the gap between clinical environments where therapies take place and the real world in which a person experiences their symptoms.^14^

Advances in virtual reality technology have given rise to novel approaches that can reduce this gap, invoking a greater sense of presence, and that aim to show greater therapeutic effectiveness.^15^ Virtual reality-based therapies (VRT) have the benefit of being able to leverage immersive, structured environments alongside gamified elements to be more engaging to people, targeting key barriers to treatment such as lack of motivation.^14,16^ Although virtual reality devices have historically been expensive, there are other advantages such as the potential to automate and digitally distribute treatment with little added cost once the treatment is developed. Access has become increasingly widened by falling hardware costs and innovations in delivery, such as formats using existing hardware like smartphones to deliver a virtual reality experience.^17^

There is no consensus on the definition of virtual reality technologies, reflecting the evolving nature of technology. As wearable headsets have become more refined, it has become necessary to draw a distinction from older concepts of virtual reality. The literature will refer to three-dimensional virtual environments delivered on traditional static computer monitors as constituting a virtual reality environment; Bisso et al refer to this modality as non-immersive or interactive virtual reality as opposed to immersive virtual reality.^18^ For the purposes of this review, virtual reality is defined as an immersive, simulated, digital experience delivered through a head-mounted display.^18,19^ In line with the prevailing distinction between these two groups, we chose to be more selective in defining virtual reality. This was done to improve the overall homogeneity of approaches tested, which can be more informative for both clinicians and software designers who might consider adopting or developing head-mounted virtual reality approaches.

There has been a notable increase in the use of digital interventions in people with SMI,^14^ and those with schizophrenia specifically.^20^ Since the review by Bisso et al in 2020, we have identified at least 12 subsequent randomised controlled trials (RCTs) that have been published, more than doubling the number of studies that we included from before this period. The increased interest in VRT is possibly driven by improvements in technology with cheaper, more powerful devices being more accessible and having a higher degree of simulation fidelity. Some steps have been taken towards automating the role of the therapist in interventions,^21^ a step that could vastly improve accessibility by enabling treatment to be delivered in the home at the individual’s convenience. However, the evidence for efficacy of automated VRT remains in its early stages.^22^ Given the pace of change and vast expansion in published trials, it is more than timely to undertake a new review of the evidence base to inform both clinical practice and further research efforts.

The aim of this study was to systematically review and assess the efficacy of virtual reality-based interventions on various health outcomes among people living with schizophrenia. Specifically, this study aims to answer the following research questions: (a) are virtual reality interventions more effective than traditional, therapeutic approaches in improving health-related outcomes for people with schizophrenia? And (b) which factors, such as intervention duration, type of virtual reality technology and patient characteristics, may influence the effectiveness of virtual reality treatment on health-related outcomes?

## Method

The review was registered with the International Prospective Register of Systematic Reviews (PROSPERO: CRD42023470849) to March 2025 prior to commencement of data extraction, and followed the guidelines for the Preferred Reporting Items for Systematic reviews and Meta-Analyses (PRISMA).^23^

We selected studies related to virtual reality-based interventions for psychotic spectrum disorders using a comprehensive search strategy. The search was conducted, from inception to the date of data extraction, using the following databases: PubMed, PsychINFO, EMBASE, CINAHL and Cochrane Central Register of Controlled Trials. Standard searches were performed using multiple keywords related to ‘virtual reality’, in combination with domains related to ‘psychosis’, ‘health outcomes’ and ‘treatment’. The full PubMed search strategy and all keywords (searched as keywords within titles, abstracts and Medical Subject Headings terms) are provided in the supplementary material. Examples include ‘VR’, ‘psychotherapy’, ‘social function’, ‘employment’, ‘sleep’, ‘psychosis’, ‘major mental illness’, ‘bipolar affective disorder’, ‘schizophrenia’ and ‘schizoaffective disorder’.^14,24^ Although we aimed to include all types of SMI in our search terms, our 15 included studies focused on a narrower diagnostic group, namely people with schizophrenia or psychotic illnesses, and our review reflects this focus. Reference lists were also hand-searched to identify any potential additional articles, and key researchers were contacted regarding unpublished data-sets. As discussed previously, we chose to include only examples of immersive virtual reality, because the degree of immersion is thought to be a key advantage of the medium.^14,24^

The titles and abstracts of potential studies identified through the initial electronic search were reviewed by two authors (G.A.C. and K.K.L.N.) independently. The initial search was conducted on 30 April 2024, followed by an update on 23 February 2025. A full-text review was conducted independently by the same authors. Any discrepancies at both levels were resolved by either a third member of the team (U.A.) or consensus by the entire review team.

The PICO framework was used to develop the eligibility criteria for this study. We intended to examine the effectiveness of any, in whole or in part, virtual reality intervention compared with traditional interventions, on adults with SMI, including schizophrenia spectrum disorders (SSDs), bipolar disorder and affective disorders with psychotic features.

The inclusion criteria were: (a) adults aged 18–65 years; (b) diagnosis of schizophrenia spectrum, bipolar or affective disorder with psychotic features; (c) any intervention delivered in whole or in part using virtual reality as a modality; (d) virtual reality-based interventions compared against interventions delivered through traditional modalities, including telehealth; (e) reporting data on any health outcome; (f) randomised or clinically controlled trials; and (g) publications in English language.

The exclusion criteria were: (a) primary diagnosis of substance use disorder; (b) populations with non-psychotic mood disorder, or bipolar affective disorder without psychotic features; (c) studies evaluating the assessment capacity of virtual reality modalities without examining any pre/post parameters; and (d) trials without a control group. Studies were excluded if the intervention was primarily a diagnostic assessment, or if the data were purely observational or qualitative.

This study did not require ethical approval because it is a systematic review of previously published studies.

Two authors (G.A.C. and K.K.L.N.) independently extracted data into an electronic spreadsheet, starting 19 June 2024 and concluding 20 November 2024. Extraction of data for the updated search started on 24 February 2025 and concluded on 10 March 2025. Disagreements were resolved by joint examination or consensus by the entire research team.

Data extracted included information at the completion of intervention and any relevant reported data from follow-up. Extracted data included baseline demographic information, dependent variables at baseline, completion and follow-up, where applicable. All data were mean and standard deviation end-point ratings, as opposed to score changes. The primary outcome measure was psychotic symptoms, affective symptoms, social functioning, cognitive symptoms, anxiety symptoms and measures of effect. Secondary outcomes included feasibility, acceptability, side-effects and measures of effect of the same.

The following data were also extracted: study location, study type, clinical setting, country of study, duration of participation, dates and length of follow-up, mean ages, ethnicity, number of participants in each group, percentage of males in each group, number of participants completed the study, duration of schooling, duration of illness in years, employment status, diagnostic criteria used, diagnosis, medication classes and different virtual reality interventions, such as virtual reality-based CBT or virtual reality-based memory training via semantic encoding.

The methodological quality of the included studies was evaluated using the Joanna Briggs Institute (JBI) Risk of Bias Tool.^25^ This tool evaluates potential biases in several domains, including randomisation, blinding, allocation concealment, outcome measurement and handling of incomplete data. The risk of bias was classified as either high or low for each domain within each study. Studies were then dichotomised into high- and low-quality categories based on their overall risk of bias, with those studies scoring >70% being considered low risk of bias and high quality.

### Statistical analysis

All data analysis was conducted in R version 4.4.0 for macOS (R Foundation for Statistical Computing, Vienna, Austria; https://www.R-project.org/). To examine the efficacy of virtual reality interventions, pre–post data – comparing baseline with end-point, and baseline with follow-up data – were converted into a common effect size (Hedges’ *g*) using the ‘esc’ package version 0.5.1 (Lüdecke D; https://doi.org/10.5281/zenodo.1249218), after which a random-effects meta-analysis was performed using the restricted maximum likelihood method.^26^ Hedges’ *g* was chosen due to its ability to provide a more accurate estimate of effect size, for smaller sample sizes, compared with odds ratio and relative risk, which were planned in our pre-published protocol. Furthermore, odds ratio and relative risk are effect sizes for binary outcomes, so Hedges’ *g* was chosen because it is able to deal with continuous effect sizes. An additional change from the protocol was that we were unable to conduct subgroup analysis by primary outcome, study quality, study duration or the use of AVATAR Therapy, due to outcomes being reported in studies with high heterogeneity of these aspects. Outcomes required *k* studies ≥3 to be meta-analysed.^27^ Publication bias was assessed using the Egger’s intercept (if *k* studies ≥10) and via the visual inspection of funnel plots (if *k* studies <10).^28^ Heterogeneity was assessed using the *I* ^2^ statistic (with >50, 51–75 and >75% being respectively deemed as low, moderate and high heterogeneity^29^) and also Cochrane’s *Q* test,^30^ with a significant test result indicating high heterogeneity. To determine the robustness of results, several sensitivity analyses were conducted. First, all meta-analyses were also conducted using the one-removed method. Second, all meta-analyses were conducted using the Knapp–Hartung adjustment, which provides more robust confidence intervals (and therefore *p*-values) where the number of studies in each analysis is limited. The credibility of results was classified according to the GRADE criteria,^31^ based on guidelines proposed by Schünemann et al,^32^ noting that only significant results were graded.

## Results

**Fig. 1:**
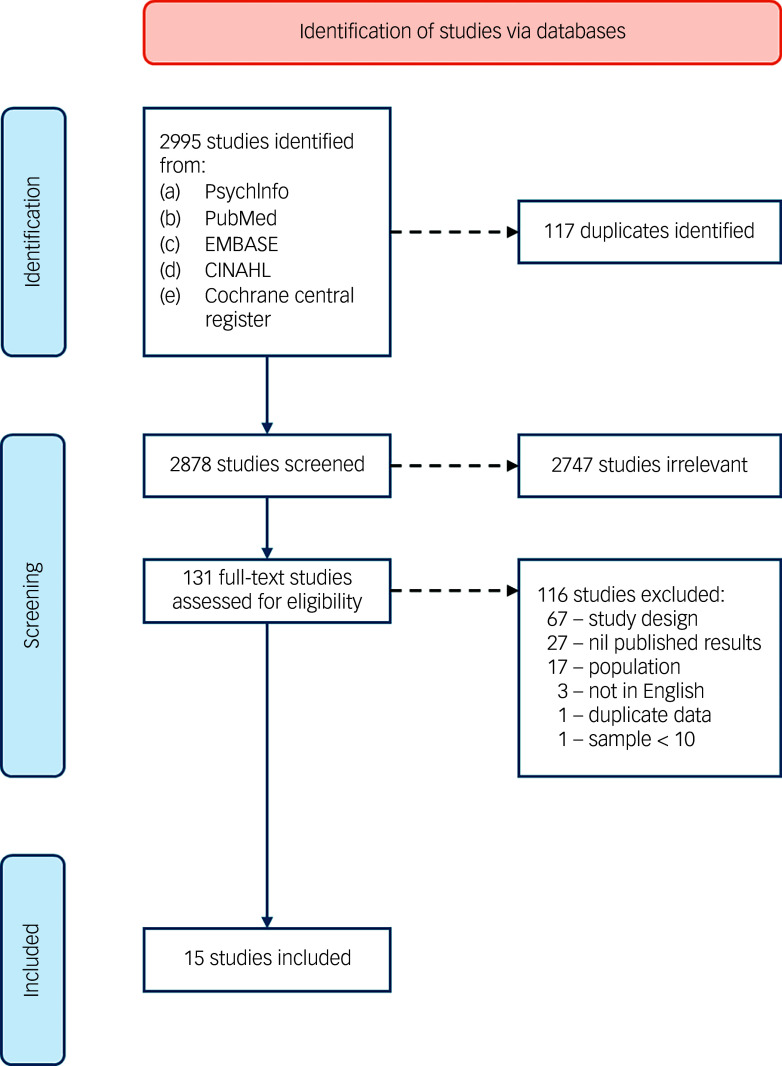
Fig. 1 long description. Preferred Reporting Items for Systematic reviews and Meta-Analyses flowchart.

The search resulted in 2878 unique studies after automatic removal of duplicates. Following screening of titles and abstracts, 2746 were excluded, with 132 included in the full-text review. Of these 132 studies, 15 met the eligibility criteria with 9 included in meta-analysis, because the other 6 did not report on an outcome that would be included in at least 3 separate studies. The PRISMA diagram is provided in Fig. 1, with details of excluded studies.

### Study characteristics

Table 1 outlines the characteristics of the 15 included studies. These studies were published between 2018 and 2025 and originated from various regions, including Europe,^33–39^ North America^40,41^ and Asia.^42,43^ Most studies recruited subjects based on DSM-IV, DSM-5 or ICD-10 criteria, except for two that used International Neuropsychiatric Interview Plus (MINI) without specifying the diagnostic criteria used,^36,44^ and three^40,42,45^ that did not specify the diagnostic criteria or diagnostic tool used.

**Table 1:** Demographic details of included studies Table 1 long description.

Study	Duration of intervention + follow-up	Country	Study setting	Diagnostic tool	Diagnosis	Primary outcome measured	Description of intervention	Control group	Mean age, years (s.d.) (control/virtual reality)	*N* (control/ virtual reality)	Percentage completed (control/virtual reality)	Male gender (control/virtual reality)
Berkhof et al^33^	12 weeks + 6 months’ follow-up	The Netherlands	Community	DSM-IV	Psychotic disorder(s)	GPTS	Virtual reality-based CBT + TAU	TAU	40.2 (9.9)/38.2 (10.1)	53/42	91/72	70/70
Bogie et al^44^	Single 15 min session	Canada	Community	Mini International Neuropsychiatric Interview	Schizophrenia or schizoaffective disorder	Hopkins Verbal Learning Test – Revised	Virtual reality-based memory training via semantic encoding	Virtual reality-based visuospatial puzzles	34.1 (11.13)/36.6 (7.58)	15/15	100/100	87/80
Cella et al^45^	12 weeks	England	Community	Unknown	A documented episode of psychosis (e.g. first-episode psychosis) and/or a diagnosis of schizophrenia	Goal Attainment Scale	Virtual reality-supported therapy for negative symptoms of psychosis + TAU	TAU	38.2 (12.5)/35.7 (9.1)	15/15	93/87	73/67
Dellazizzo et al^40^	9 weeks + 12 months’ follow-up	Canada	Community	Unknown	Treatment-resistant schizophrenia or schizoaffective disorder	PSYRATS-AH	Virtual reality-assisted therapy	CBT + TAU	41.4 (13.4)/43.6 (12.0)	37/37	92/76	73.0/78.4
Du Sert et al^41^	7 weeks + 3 months’ follow-up	Canada	Community	DSM-5	Treatment-resistant schizophrenia or schizoaffective disorder	PSYRATS-AH	Virtual reality-assisted therapy	TAU	42.9 (12.4)^a^	15^a^	79^a^	66.7^a^
Li et al^43^	2 weeks	China	Hospital	ICD-10	Schizophrenia (remission)	MCCB	Virtual reality-based cognitive training	TAU	47.5 (unknown)/46.0 (unknown)	32/30	94/88	59/67
Liang et al^42^	9 weeks + 3 months’ follow-up	China	Community	Unknown	Treatment-resistant schizophrenia	PSYRATS-AH	Virtual reality-based computer AT system	CBT	26.5 (6.8)/25.3 (5.3)	33/32	85/94	52/44
Nijman et al^36^	8 weeks + 3 months’ follow-up	The Netherlands	Community	Mini International Neuropsychiatric Interview Plus	Psychotic disorders	Social cognition and social functioning	Dynamic interactive social cognition training in virtual reality	Virtual reality relaxation	39.7 (12.4)/35.9 (10.4)	40/41	78/61	65/73
Park et al^37^	5 weeks	Korea	Hospital	DSM-IV	Schizophrenia	Social skills – Trower’s Social Behaviour Scales	Virtual reality social skills training	Non-virtual reality/traditional social skills training	31.2 (7.7)/28.1 (7.7)	37/39	84/85	58/48
Pot-Kolder et al^34^	8–12 weeks + 6 months’ follow-up	The Netherlands	Community	DSM-IV	Psychotic disorders	Social participation	Virtual reality-based CBT + TAU	TAU (waiting list control)	39.5 (10)/36.5 (10)	58/58	91/86	72/69
Shen et al^35^	10 sessions	China	Hospital	DSM-5	Schizophrenia	Social function and social cognition via various scales	Virtual reality social cognition and interaction training (SCIT) + TAU	Non-virtual reality/traditional SCIT + TAU	30.7^b^ (6.6)/31.0^c^ (7.5)/30.8 (4.9)	29^b^/30^c^/28	93^b^/77^c^/90	67^b^/65 ^c^/65%
Van der Stouwe et al^46^	6 months	The Netherlands	Community	DSM-IV	Psychotic disorders	Experience sampling (daily life stress, negative affect, paranoia)	Virtual reality CBT	Waiting list control	39.5 (10)/36.5 (10)	58/58	84/81	72/69
Vass et al^39^	9 weeks	Hungary	Community	DSM-IV-TR	Schizophrenia	PANSS	Virtual reality-based targeted theory of mind (VR-ToMIS)	Passive virtual reality + TAU	48.8 (8.9)/38.6 (13.5)	9/8	100/100	38/56
Vass et al^38^	9 weeks	Hungary	Community	DSM-5	Schizophrenia or schizoaffective disorder	PANSS	VR-ToMIS	Passive virtual reality	42.5 (8.74)/36.7 (11.7)	21/21	100/95	48/57
Wang et al^47^	At least 10 days	China	Hospital	ICD-10	Schizophrenia	Cognitive function	Virtual reality serious game training programme + TAU	TAU	26.9 (6.3)/24.6 (5.7)	33/31	100/100	52/45

This table provides an overview of the study characteristics, interventions and population demographics of the included studies. CBT, cognitive–behavioural therapy; TAU, treatment-as-usual; GPTS, Green et al. Paranoid Thoughts Scales; PSYRATS-AH, Psychotic Symptom Rating Scales – Auditory Hallucination; MCCB, MATRICS (Measurement and Treatment Research to Improve Cognition in Schizophrenia) Consensus Cognitive Battery; AT, avatar therapy; PANSS, Positive and Negative Syndrome Scale.

a. Not distinguished between control and intervention groups.

b. TAU.

c. Traditional SCIT.

Although ethnicity data were extracted where reported, the included studies largely comprised ethnically homogeneous samples, typically reflecting a single dominant ethnic group within the country of recruitment. Reporting of ethnicity was inconsistent, and representation of ethnically diverse or minority populations was limited.

The included virtual reality interventions across the studies varied in format. Most were 45–60 min sessions delivered over 6–16 sessions, typically weekly. A single study focused on cognitive function, conducting virtual reality intervention once per day, 5 days per week for 2 weeks in total.^43^ Whereas most interventions used virtual reality–CBT frameworks,^33,34,40^ other studies simulated everyday social environments (e.g. public transport, supermarkets, cafes) to address paranoia, social anxiety and cognitive deficits.^38,39,43^ All interventions were guided by a therapist either during or following the virtual reality exposure, which ranged from direct in-session guidance to post-session debriefing. Overall, interventions blended immersive technology with psychological principles to enhance real-world functioning in psychosis.

Participants were recruited from both in-patient (*n* = 289) and out-patient settings (*n* = 681) – 4 and 11 studies, respectively. Sample size varied across studies, ranging from 15 to 116 participants.^34^ Whereas most studies included individuals with schizophrenia or schizoaffective disorder, some focused specifically on people with treatment-resistant schizophrenia.^40,42^ One study examined the effects of a virtual reality intervention on people in the remission stage.^43^

The nature of virtual reality-based interventions varied across studies. A virtual reality environment was used to deliver assisted therapy,^33,34,40,41^ a cognitive training system,^43^ social cognition training,^36^ computer AVATAR therapy^42^ and Theory of Mind intervention.^38,39^ Control conditions differed among studies, including treatment-as-usual (TAU), supportive therapy, traditional CBT, relaxation-based virtual reality interventions and passive virtual reality conditions, in which no intervention was delivered. Intervention duration ranged from 2 weeks^43^ to 3 months.^33,34^

Outcome measures used were largely heterogeneous. Six studies reported outcomes using the Positive and Negative Syndrome Scale total (PANSS total); a further six studies reported the negative PANSS subscale, five reported the positive PANSS subscale and four reported the general PANSS subscale. Four studies reported depression using the Beck Depression Inventory (BDI) and three reported paranoia using Green et al. Paranoid Thoughts Scales (GPTS)-A. Many other preplanned outcomes on social functioning, cognitive symptoms, feasibility, acceptability and side-effects could not be undertaken because insufficient data were available for these outcomes. These include, but are not limited to, measures of Measurement and Treatment Research to Improve Cognition in Schizophrenia consensus cognitive battery,^43^ social cognition, social functioning, neurocognitive measures^36^ and quality of life.^41^

### Quality assessment

Five studies^33–36,40,43^ were rated as high quality, with a low risk of bias in most domains. The remaining studies^38,39,41,42^ had a moderate risk of bias due to limitations in allocation concealment, blinding procedures and incomplete follow-up data.

### Meta-analyses

#### Effects of virtual reality interventions from baseline to end-point

**Fig. 2:**
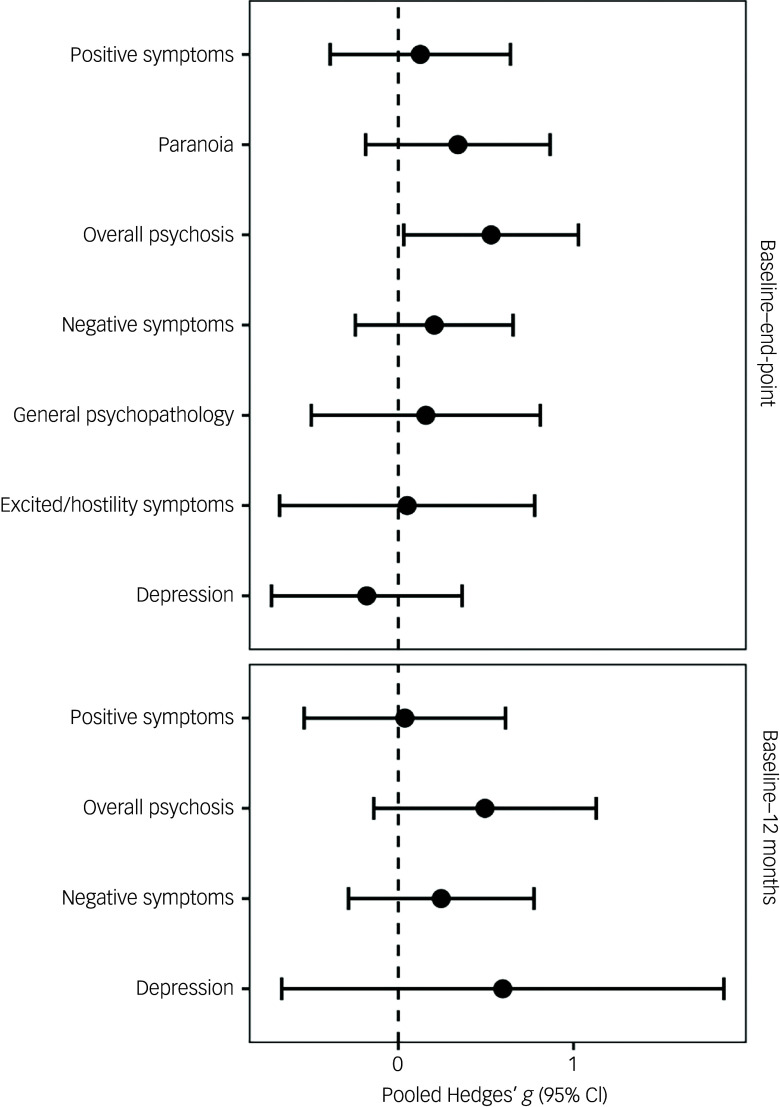
Fig. 2 long description. Summary of meta-analyses (baseline to end-point).

**Fig. 3:**
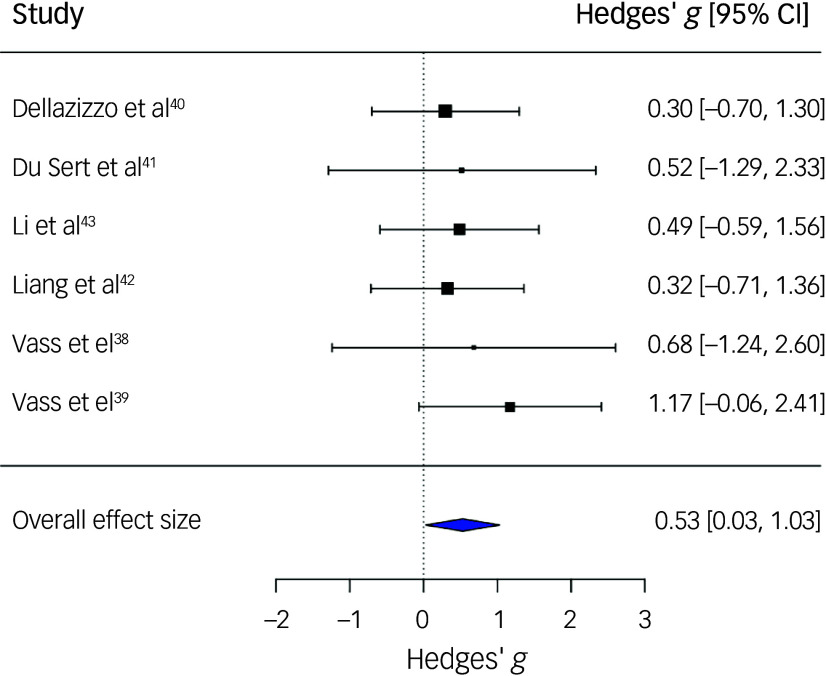
Fig. 3 long description. Forest plot for Positive and Negative Syndrome Scale total score (baseline to end-point).

The results of meta-analyses assessing the impact of virtual reality interventions on all outcomes, using effect sizes from baseline to end-point, are summarised in Figs 2 and 3 and Table 2. Virtual reality interventions were effective at reducing overall psychosis symptoms (Hedges’ *g* = 0.53, 95% CI [0.03, 1.03], *p* = 0.037, *k* = 6). This result received a GRADE rating of ‘very low’ certainty due to substantial imprecision and indirectness. No other outcomes yielded significant results (see Table 2).

**Table 2:** Baseline to end-point meta-analysis results Table 2 long description.

Outcome	*k* studies	*N* participants (*n* intervention; *n* control)	Meta-analysis		Heterogeneity		Level of certainty
			Hedges’ *g* (95% CI)	*P*-value	*I* ^2^ (%)	Cochrane’s *Q* (*p*-value)	
Overall psychosis symptoms	6	263 (134; 129)	0.53 (0.03; 1.03)	0.037	0	1.43 (0.922)	Very low
Positive psychosis symptoms	6	314 (159; 155)	0.13 (−0.39; 0.64)	0.632	0	0.90 (0.924)	NS
Negative psychosis symptoms	6	314 (159; 155)	0.21 (−0.25; 0.66)	0.372	0	1.93 (0.859)	NS
General psychopathy	3	210 (109; 101)	0.16 (−0.50; 0.81)	0.637	0	1.39 (0.499)	NS
Depression	4	214 (107; 107)	−0.18 (−0.72; 0.36)	0.516	0	1.01 (0.800)	NS
GPTS-A	3	225 (105; 120)	0.34 (−0.19; 0.87)	0.206	0	0.98 (0.612)	NS
Excited psychosis symptoms	3	121 (59; 62)	0.05 (−0.68; 0.78)	0.892	0	0.70 (0.705)	NS

NS, not significant; GPTS-A, Green et al. Paranoid Thoughts Scale – Part A.

For individual symptom domains, no significant effects were observed for positive symptoms (Hedges’ *g* = 0.13, 95% CI [−0.39, 0.64], *p* = 0.632, *k* = 6), negative symptoms (Hedges’ *g* = 0.21, 95% CI [−0.25, 0.66], *p* = 0.372, *k* = 6), general psychopathology (Hedges’ *g* = 0.16, 95% CI [−0.50, 0.81], *p* = 0.637, *k* = 3), depression (Hedges’ *g* = −0.18, 95% CI [−0.72, 0.36], *p* = 0.516, *k* = 4), GPTS-A (Hedges’ *g* = 0.34, 95% CI [−0.19, 0.87], *p* = 0.206, *k* = 3) or excited psychosis symptoms (Hedges’ *g* = 0.05, 95% CI [−0.68, 0.78], *p* = 0.892, *k* = 3). No evidence of publication bias was detected in the primary meta-analysis. Funnel plots did not indicate asymmetry (see supplementary materials for details).

#### Effects of virtual reality interventions from baseline to 12-month follow-up

As with individual symptom domains from baseline to end-point, the baseline to 12-month follow-up analysis revealed no significant results on any outcome (see Table 3). Total overall psychosis symptoms at 12 months were not significantly different between intervention and control groups (Hedges’ *g* = 0.50, 95% CI [−0.14, 1.13], *p* = 0.127, *k* = 3). No significant effects were found for positive symptoms (Hedges’ *g* = 0.04, 95% CI [−0.54, 0.61], *p* = 0.899, *k* = 4), negative symptoms (Hedges’ *g* = 0.25, 95% CI [−0.28, 0.77], *p* = 0.364, *k* = 4) or depression (Hedges’ *g* = 0.60, 95% CI [−0.67, 1.86], *p* = 0.354, *k* = 3).

**Table 3:** Baseline to 12-month follow-up meta-analysis results Table 3 long description.

Outcome	*k* studies	*N* participants (*n* intervention; *n* control)	Meta-analysis		Heterogeneity		Level of certainty
			Hedges’ *g* (95% CI)	*P*-value	*I* ^2^ (%)	Cochrane’s *Q* (*p*-value)	
Overall psychosis symptoms	3	155 (78; 77)	0.50 (−0.14; 1.13)	0.127	0	0.63 (0.732)	NS
Positive psychosis symptoms	4	222 (111; 111)	0.04 (−0.54; 0.61)	0.899	0	0.73 (0.867)	NS
Negative psychosis symptoms	4	222 (111; 111)	0.25 (−0.28; 0.77)	0.364	0	1.17 (0.760)	NS
Depression	3	181 (87; 94)	0.60 (−0.67; 1.86)	0.354	77.3	8.36 (0.015)	NS

NS, not significant.

### Sensitivity analyses

Sensitivity analyses demonstrated that the removal of either the study of Vass et al^38^ or Li et al^43^ led to a loss of statistical significance for total psychotic symptoms. The removal of any one study did not alter the significance, direction or magnitude of any other outcomes (see supplementary materials). Use of the Hartung−Knapp adjustment did not alter the significance or magnitude of results.

## Discussion

Our key findings are as follows: (a) people treated with VRT were more likely to experience an overall reduction in their symptoms of schizophrenia (as measured by PANSS) than those treated with passive or active controls; (b) however, compared with control condition, VRT did not improve any subgroup of psychotic symptoms, nor symptoms of depression or paranoia; and (c) there were no significant differences in paranoid ideation. Taken together, these findings indicate that VRT are generally feasible and potentially effective at reducing overall symptoms of psychosis. In practice, this suggests that VRT may subtly improve multiple symptom domains in a way that produces a larger and more appreciable effect size on total scores than on individual subgroups.

These findings, however, must be interpreted with significant caution. The small number of pooled studies contributing to each comparison significantly limits the statistical power of our analysis. As such, our results cannot be interpreted with any degree of certainty and should be regarded as exploratory rather than confirmatory. Despite this limitation, our meta-analysis remains informative in the context of a sparse and clinically heterogeneous evidence base because it enables a transparent synthesis of available data, highlights the imprecision surrounding current analysis and provides a more interpretable framework than a narrative summary alone.

There are several previous literature reviews on the use of virtual reality in people with SMI such as schizophrenia, although early reviews are characterised by a lack of RCTs. An initial Cochrane review in 2014 identified three trials that used virtual reality to improve occupational skills training, all in schizophrenia. They found no significant evidence in favour of virtual reality over controls, although the authors noted that small sample sizes had imparted very low certainty to the evidence.^48^ In 2016 Valmaggia et al,^19^ with a broader research question, identified 24 studies on the use of virtual reality to treat mental illness. They identified two trials that showed positive evidence for the use of virtual reality in treating social and cognitive function, as well as in reducing negative symptoms in people with schizophrenia. The group also included two further studies showing that virtual reality interview training improved interview performance, compared with TAU, for people with schizophrenia.

Subsequently, Bisso et al reported more mixed evidence on the effectiveness of virtual reality social skills training in people with SSDs.^18^ This group identified five trials showing the efficacy of VRT in treating positive symptoms (delusions, paranoia, auditory verbal hallucinations), and in improving cognition. Similarly, a review by Freeman et al also noted evidence for virtual reality treatment of positive symptoms, identifying six trials with positive results despite small group sizes.^49^

Since this review was commenced there has been further work published with similar goals. Spark et al included seven studies with some cross-over with our sample.^50^ In their analysis of six studies, the team averaged the overall effect size of different outcome measures within studies and grouped these by symptom type, i.e. scales measuring delusions. These averages were then compared across studies, and the group showed that VRT were associated with improvements in scales assessing delusional symptoms of schizophrenia. Bell et al reviewed the field with a more thematic approach, characterising the strengths, weaknesses and recent developments of the current literature.^14^ The current heterogeneity in methodological approaches presents a dilemma for designing reviews on VRT. Broad inclusion criteria carry a risk of aggregating studies with limited comparability, whereas more restricted criteria improve internal consistency but reduce the pool of included studies, impairing statistical power.

In summary, the existing literature suggests that there is stronger, yet inconclusive, evidence in favour of VRT in treating positive symptoms compared with social skills or negative symptoms.^48^

Surprisingly, our analysis showed no significant evidence of VRT being more effective at treating positive symptoms. This is contrary to the results of previous reviews, and to the findings of individual studies that focused on this subgroup of symptoms. In reviewing the studies that aimed to improve social or cognitive outcomes with VRT, it is noticeable that these do not show much, if any, effect on positive symptom scales. As such, it is possible that this subgroup of papers masks the positive findings of other studies. Contrary to the findings of Valmaggia et al,^19^ we did not find significant evidence of virtual reality effectively improving negative symptoms, but this is perhaps explained by the relative focus of our sample on interventions targeting positive symptoms or social cognition. Our outcome was somewhat in keeping with previous reviews that identified evidence suggestive of VRT being effective at treating a range of symptoms of schizophrenia.^18,49^

Taken together, our results are relatively positive regarding the efficacy of VRT, especially considering that even when comparing vastly different approaches there appears to be a significant overall benefit to general symptoms. There is clearly a strong narrative around VRT based on AVATAR and cognitive behavioural therapy for psychosis (CBTp) approaches, so it is noteworthy that this is not reflected in our results. There are probably several factors at play, not least that the number of trials contributing to our comparison remains relatively small. One hypothesis is that interventions that give patients a greater sense of control over their auditory verbal hallucinations (AVH) are likely to reduce their anxiety, and this is subsequently reflected in lower general psychopathology scores. We did not find any direct evidence of this, because only three studies reported on this subscale separately. It is possible that data from social–cognitive focused on studies that mask the positive symptom scale data of studies targeting positive symptoms.

There was some evidence that both the presence and severity of safety behaviours, which function to reduce feelings of perceived threat while maintaining maladaptive beliefs, influence the effectiveness of VRT in treating positive symptoms. One study in particular identified safety behaviours as a mediator of treatment response.^34^ Moderator analysis of all three groups that compared virtual reality−CBT against TAU^33,34,46^ found that levels of safety behaviours were a significant variable associated with greater reductions in paranoia. These findings appear to be in keeping with the results of Freeman et al,^51^ indicating that VRT are more effective for people with more severe symptoms of agoraphobic avoidance and who also have higher levels of safety behaviours. Although it is possible that the presence of safety behaviours may correlate only as a marker of severe paranoia, it is also possible that the way people socially engage with safety behaviours is mechanistically important in regard to perpetuation of their paranoia. Pot-Kolder et al^34^ suggest that VRT enable patients to drop their safety behaviours: as a result they experience positive social interactions and this effect compounds. This pattern of more severe illness responding better to VRT also corroborates the findings of two studies conducted on Chinese in-patients,^43,47^ where virtual reality-based interventions of relatively short duration (10–14 days) were associated with significant improvements in cognitive function.

Agreeing a set of core outcome measures for virtual reality trials would greatly aid their repeatability and comparability, and is of particular importance while the number of trials remains sparse. Our study identified that PANSS total scores were the most commonly used outcome measure, but even this measure was present in fewer than half of studies. Although it is appreciated that trials are designed to answer different research questions and that there is a burden to include extra assessments, the inclusion of a general symptom scoring tool like PANSS as a secondary outcome measure may be a useful standard for the literature to adopt. Finally, although the evaluation of clinical efficacy of novel digital therapeutic approaches such as virtual reality interventions is, indeed, an important research endeavour, the assessment of safety, as well as their cost–benefit value, should also be part of future virtual reality-based trials.^52^

Within our sample there was limited reporting of feasibility and safety data related to VRT. Of the 15 studies reviewed, only 3 assessed symptoms such as nausea or other adverse effects post-intervention, and only 1^44^ reported these findings in the main text. Although the findings of Bogie et al,^44^ who reported good tolerability, are encouraging, it is noteworthy that, in the analysis of completion rates across the sample (Fig. 1), only Liang et al^42^ reported higher attrition in the control group. Given the known risks associated with SMI,^53^ it is essential that any novel intervention demonstrates both safety and tolerability. International recommendations have already called for improved safety standards in digital mental health interventions, including more robust monitoring and reporting of adverse events.^52^ It is therefore hoped that future studies will provide more comprehensive data on potential adverse outcomes.

It is noteworthy that all the interventions tested in the studies included in this review required face-to-face human support. This may reflect both the complexity of the intervention and the nature of the population with its symptoms and, especially given the nature of positive psychotic symptoms (e.g. hallucinations and paranoid symptoms), is likely to be the most feasible way of delivering VRT. Nevertheless, such support raises the costs and limits virtual reality use to structured clinical environments. Although the optimal role of the human factor in delivering digital mental health interventions among people with SSDs such as schizophrenia remains unclear,^20^ it may not always be a requirement that a human is physically or digitally present. Automating therapy, such as via virtual therapists, may reduce costs as well as improve accessibility.^21^ Nevertheless, it is important to highlight the implicit safety risks of reducing or abolishing the human factor, and that these must be fully characterised, as well as consulted with the end-users,^54^ before this technology can become widespread.

Compounding the above safety concerns is the rapid pace of change that artificial intelligence-driven virtual reality interventions are already bringing to other areas of healthcare.^55^ Although the transformative potential of artificial intelligence is evident, it is critical to develop a thorough understanding of both the benefits and risks associated with virtual reality interventions, particularly for people with schizophrenia, who are a highly vulnerable group. One way to achieve this is through the increased use of mixed-methods studies, which can capture participants’ personal experiences with virtual reality and enhance the recording and reporting of adverse side-effects.^54^ Such an approach would help ensure that the development and implementation of virtual reality interventions are informed by both empirical evidence and the lived experiences of users, ultimately promoting safer and more effective care for this population.

The strengths of our study lie in its furthering of our understanding of the use of VRT in a more homogenous population, specifically people with schizophrenia and related disorders. We also explored comparisons of VRT against both passive and active controls, providing insight into the comparative value of VRT against other intervention types. Our study also compared a larger number of studies than previous reviews, with sample size noted as a key limitation. The VRT included in our meta-analysis included a wide range of approaches that differed in content, technology, session length, number of sessions and facilitator expertise. Our included studies could be mostly subdivided by aim into two groups, with those aiming to improve social or cognitive function being the largest (eight), followed by papers aiming to improve distress from AVH/paranoia (six). Pooling these groups may have obscured the unique aspects of treatment designs that drive the significant findings of individual trials. Likewise, combining passive and active controls assumes that these comparators have the same therapeutic influence when, in reality, active controls may themselves yield some positive benefit. This heterogeneity in both the aim and design of our pooled sample reduces the analytical power of analysis and restricts the generalisability of our conclusions.

The lack of methodological concordance across our sample also presented a challenge to our analysis. This was particularly noticeable in the diversity of outcome measures recorded, with our group identifying over 65 distinct measures. As a result, despite the inclusion of 14 trials in our final sample, less than half contributed to individual outcome comparisons. This significantly reduced the statistical power of our analysis and increased the chance of both type 1 and 2 errors. Due to the limited number of studies contributing to individual comparisons, we were also unable to conduct subgroup and moderator analysis, impacting our assessment of whether treatment effects varied according to study characteristics.

Without stratifying by type of control group, we could not determine whether VRT out-perform active controls. It would also have been useful to determine whether there is a significant difference in effect between passive virtual reality and active VRT, because this would shed light on whether the medium itself has any intrinsic effect. Similarly, we were precluded from conducting a sensitivity analysis of potential differential effects by different stages of illness. The absence of meta-regression or moderator analysis limits our ability to evaluate the impact of other factors such as sample size, baseline symptom severity and risk of bias ratings. We aimed to offset this by providing a narrative synthesis of our included studies.

In conclusion, VRT can be an effective tool in treating the overall symptoms of schizophrenia, although there is limited evidence supporting its effectiveness in treating negative and affective symptoms of schizophrenia. Our findings highlight the need for further studies using standard validated measures to enable subgroup analysis, and indicate that improving the comparability of future studies will be crucial in proving the efficacy of VRT.

## Supporting information

10.1192/bjo.2026.12012.sm001 Colgan et al. supplementary material 1 Colgan et al. supplementary material

10.1192/bjo.2026.12012.sm002 Colgan et al. supplementary material 2 Colgan et al. supplementary material

10.1192/bjo.2026.12012.sm003 Colgan et al. supplementary material 3 Colgan et al. supplementary material

10.1192/bjo.2026.12012.sm004 Colgan et al. supplementary material 4 Colgan et al. supplementary material

10.1192/bjo.2026.12012.sm005 Colgan et al. supplementary material 5 Colgan et al. supplementary material

10.1192/bjo.2026.12012.sm006 Colgan et al. supplementary material 6 Colgan et al. supplementary material

10.1192/bjo.2026.12012.sm007 Colgan et al. supplementary material 7 Colgan et al. supplementary material

## Data Availability

Data availability is not applicable to this article because no new data were created or analysed in this study.

## Supplementary material

The supplementary material is available online at https://doi.org/10.1192/bjo.2026.12012

## Fig. 1 Long description

The flowchart begins with the identification of 2995 studies from databases including PsychInfo, PubMed, EMBASE, CINAHL and Cochrane Central Register. 117 duplicates are identified and removed. 2878 studies are screened, with 2747 deemed irrelevant. 131 full-text studies are assessed for eligibility, and 116 are excluded for various reasons such as study design, lack of published results, population issues, language, duplicate data and small sample size. Ultimately, 15 studies are included in the review.

Navigate back to Fig. 1.

## Table 1 Long description

The table presents demographic details of 15 studies on virtual reality interventions for psychotic disorders. It includes columns for study, duration of intervention and follow-up, country, study setting, diagnostic tool, diagnosis, primary outcome measured, description of intervention, control group, mean age in years, number of controls and virtual reality participants, percentage completed, and male gender percentage in control and virtual reality groups. The studies vary in duration, setting, and diagnostic criteria, with participants from Europe, North America, and Asia. The table highlights differences in study designs, participant demographics, and outcomes measured.

Navigate back to Table 1.

## Fig. 2 Long description

The image presents two horizontal box-and-whisker plots comparing pooled Hedges’ *g* values with 95 percent confidence intervals for various symptoms at two different time points: baseline to end-point and baseline to 12 months. The x-axis represents the pooled Hedges’ *g* values ranging from 0 to 1, while the y-axis lists different symptoms: Positive symptoms, Paranoia, Overall psychosis, Negative symptoms, General psychopathology, Excited/hostility symptoms, and Depression. Each box plot shows the median, lower quartile, and upper quartile values, with whiskers indicating the range of data. The first plot at the top shows data for baseline to end-point, while the second plot at the bottom shows data for baseline to 12 months. The boxes and whiskers vary in length, indicating differences in the spread and central tendency of the data for each symptom. All values are approximated.

Navigate back to Fig. 2.

## Fig. 3 Long description

A box-and-whisker plot displays the Hedges’ g values for different studies. The plot includes six individual box plots and one overall effect size box plot. The x-axis represents the studies, and the y-axis represents the Hedges’ g values ranging from -2 to 3. Each box plot shows the median, lower quartile, and upper quartile values, with whiskers indicating the range of data. The overall effect size is highlighted in blue. The studies listed are Dellazizzio et al, Du Sert et al, Li et al, Liang et al, Vass et al, and Vass et al. The overall effect size is 0.53 with a 95% confidence interval of 0.03 to 1.03. All values are approximated.

Navigate back to Fig. 3.

## Table 2 Long description

The table presents a meta-analysis of virtual reality interventions on various psychosis symptoms. It includes six studies with a total of 263 participants for overall psychosis symptoms, showing a significant reduction with a Hedges’ g of 0.53 and a p-value of 0.037. The table also covers positive psychosis symptoms, negative psychosis symptoms, general psychopathology, depression, and OPT3-A, with no significant results for these outcomes. The heterogeneity and level of certainty are also detailed, with the overall psychosis symptoms result rated as very low certainty.

Navigate back to Table 2.

## Table 3 Long description

The table presents a meta-analysis of outcomes from baseline to 12-month follow-up, comparing intervention and control groups. It includes four rows for different outcomes: overall psychosis symptoms, positive psychosis symptoms, negative psychosis symptoms, and depression. Each row lists the number of studies, participants, Hedges’ *g* with 95% confidence intervals, *p*-value, heterogeneity *I*-squared, Cochrane’s Q *p*-value, and level of certainty. The table shows no significant differences between intervention and control groups for any outcome. Overall psychosis symptoms have a Hedges’ *g* of 0.50 with a *p*-value of 0.127. Positive psychosis symptoms have a Hedges’ *g* of 0.04 with a *p*-value of 0.899. Negative psychosis symptoms have a Hedges’ *g* of 0.25 with a *p*-value of 0.364. Depression has a Hedges’ *g* of 0.60 with a *p*-value of 0.354. The heterogeneity and level of certainty vary across outcomes.

Navigate back to Table 3.
