# Supplementary material for: Do virtual reality-based therapies affect symptomatology and psychosocial functioning in schizophrenia spectrum disorders: systematic review and meta-analysis
Source: BJPsych Open. 2026 Jun 18;12(4):e165. doi: 10.1192/bjo.2026.12012 (PMC13276771; doi:10.1192/bjo.2026.12012)
Supplement: Colgan et al. supplementary material 3 — Colgan et al. supplementary material [file S2056472426120122sup003.pdf]

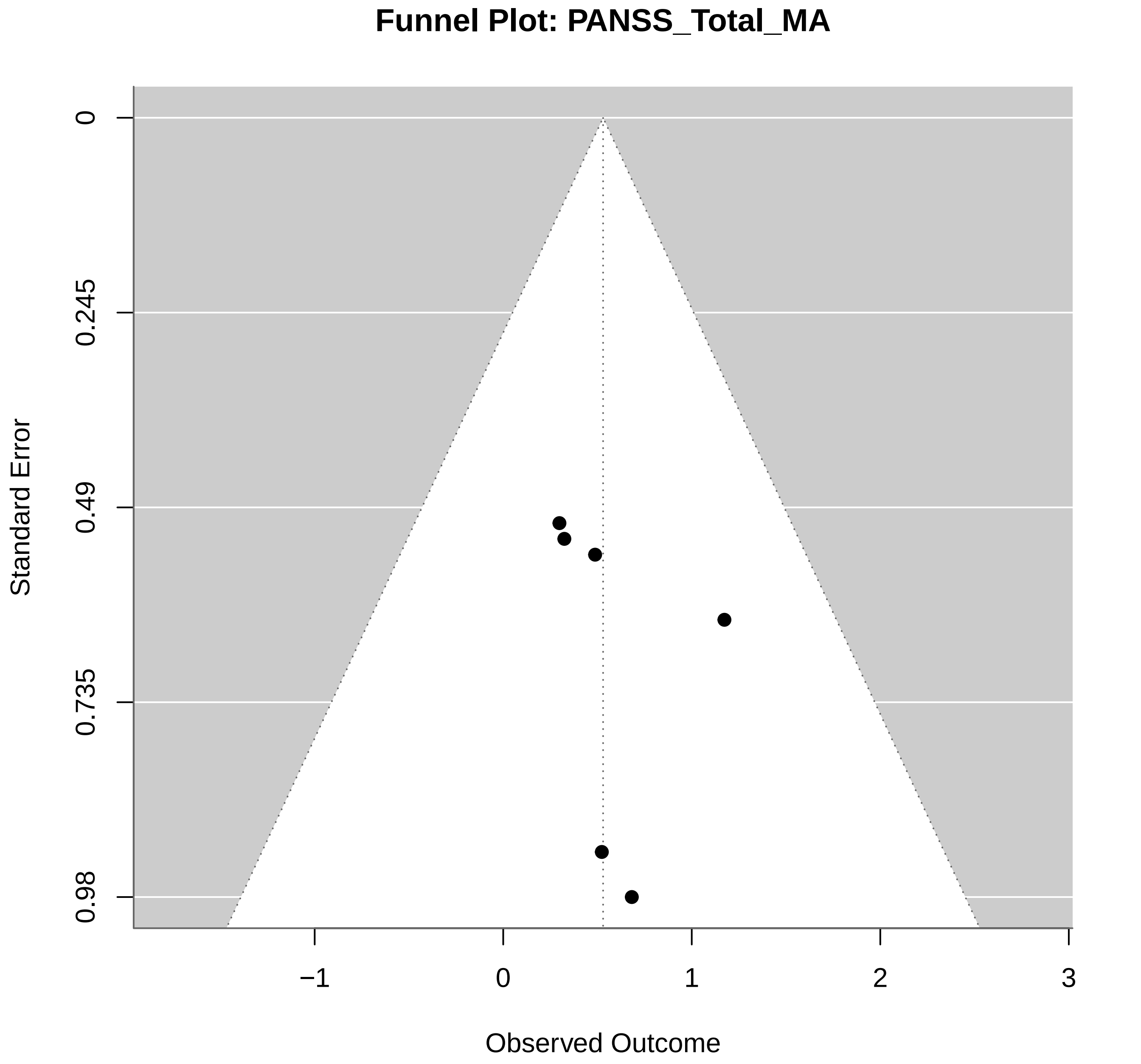

Funnel Plot: PANSS_Total_MA
Standard Error
0
0.245
0.49
0.735
0.98
−1
0
1
2
3
Observed Outcome

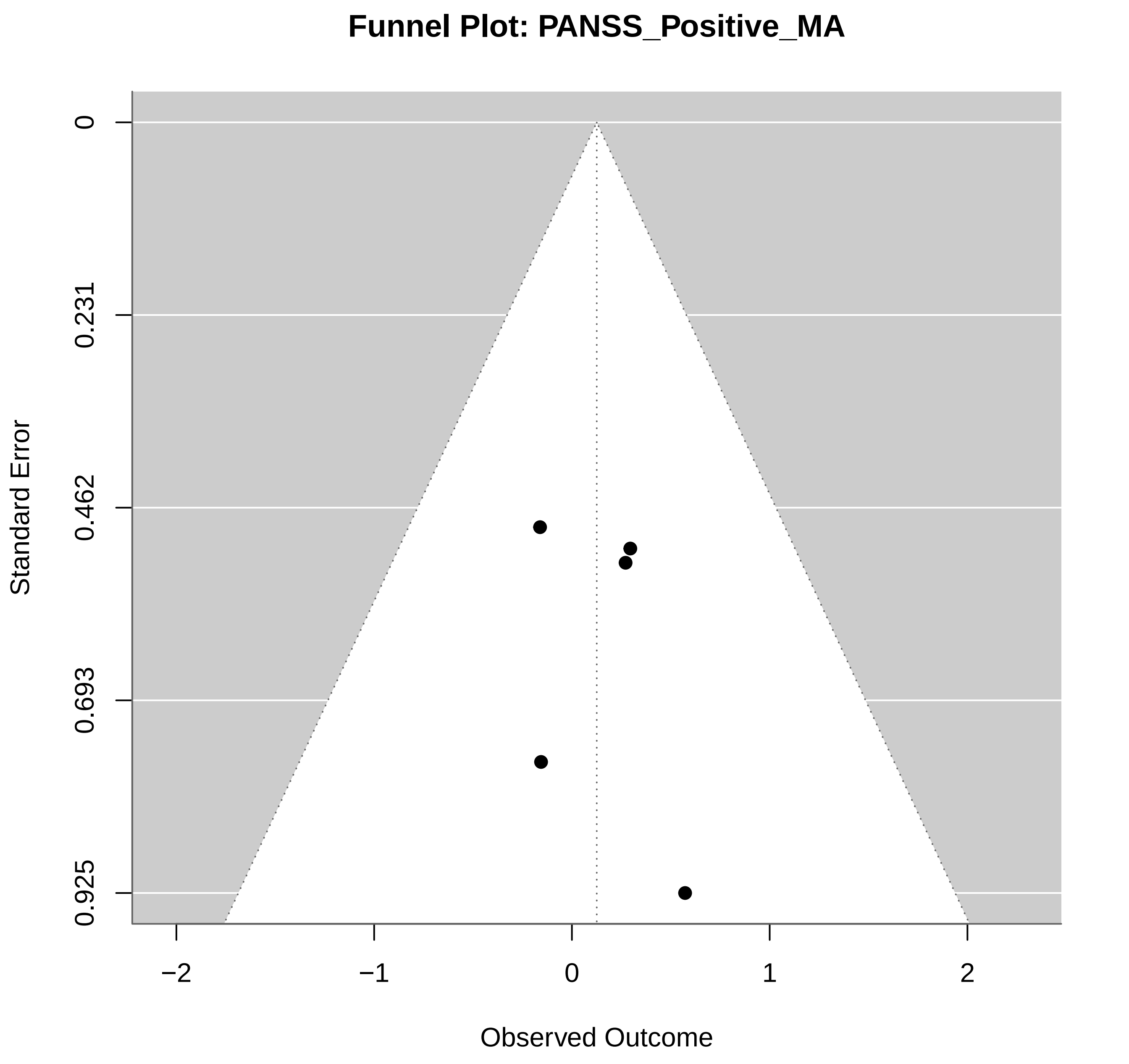

Funnel Plot: PANSS_Positive_MA
Standard Error
0
0.231
0.462
0.693
0.925
−2
−1
0
1
2
Observed Outcome

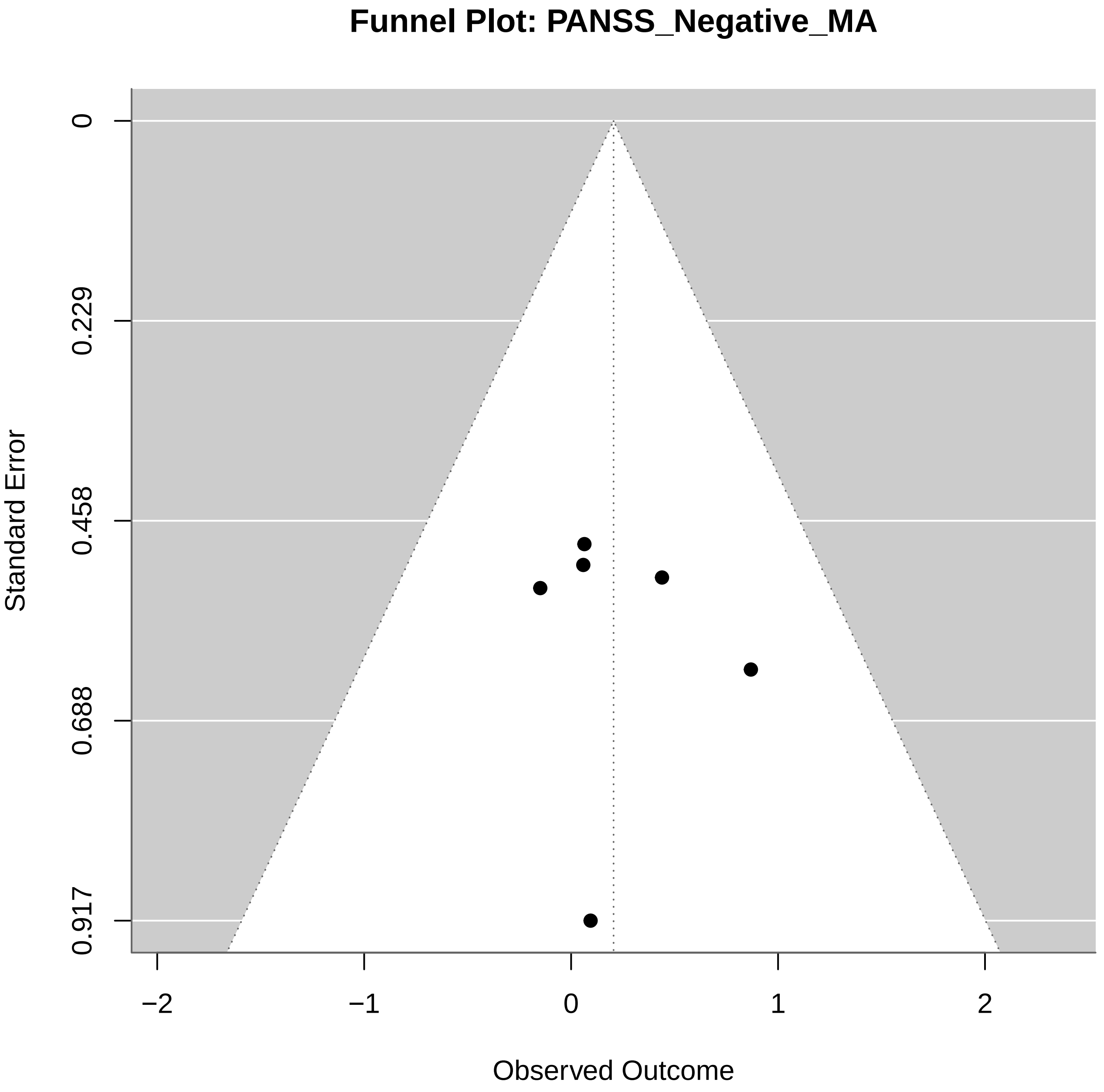
Funnel Plot: PANSS_Negative_MA
Standard Error
0
0.229
0.458
0.688
0.917
−2
−1
0
1
2
Observed Outcome

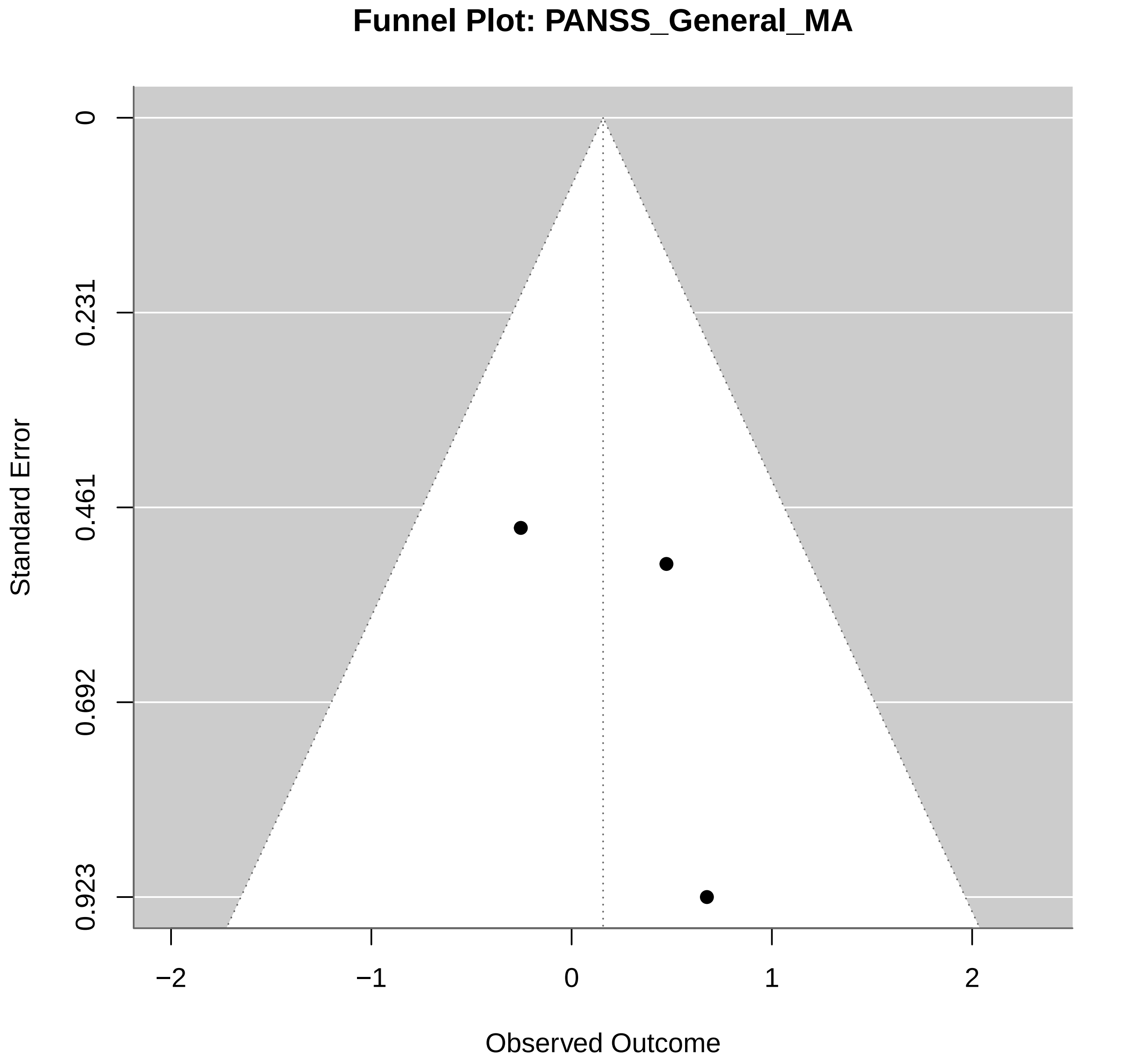

Funnel Plot: PANSS_General_MA
Standard Error
0
0.231
0.461
0.692
0.923
−2
−1
0
1
2
Observed Outcome

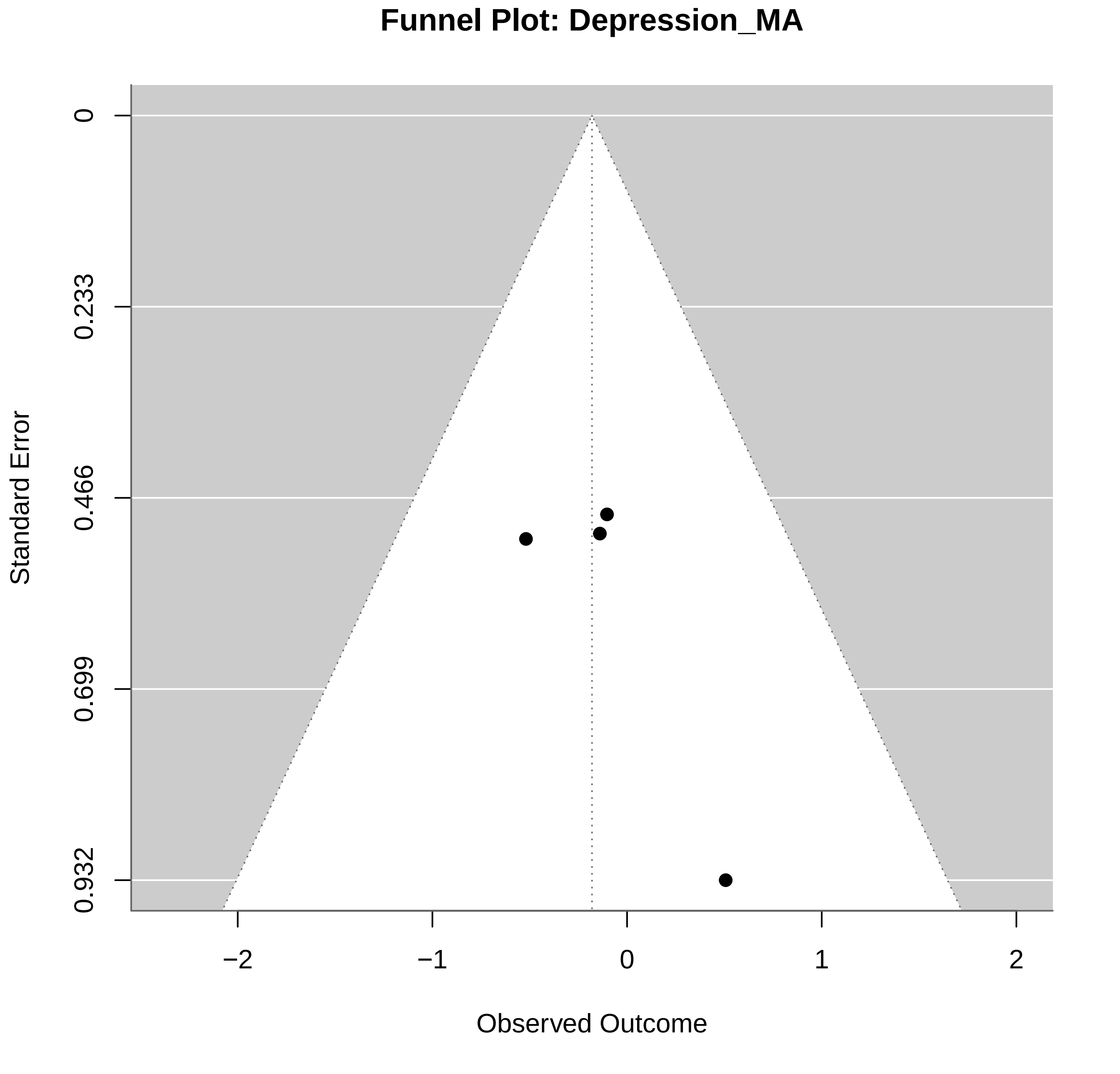
Funnel Plot: Depression_MA
Standard Error
0
0.233
0.466
0.699
0.932
−2
−1
0
1
2
Observed Outcome

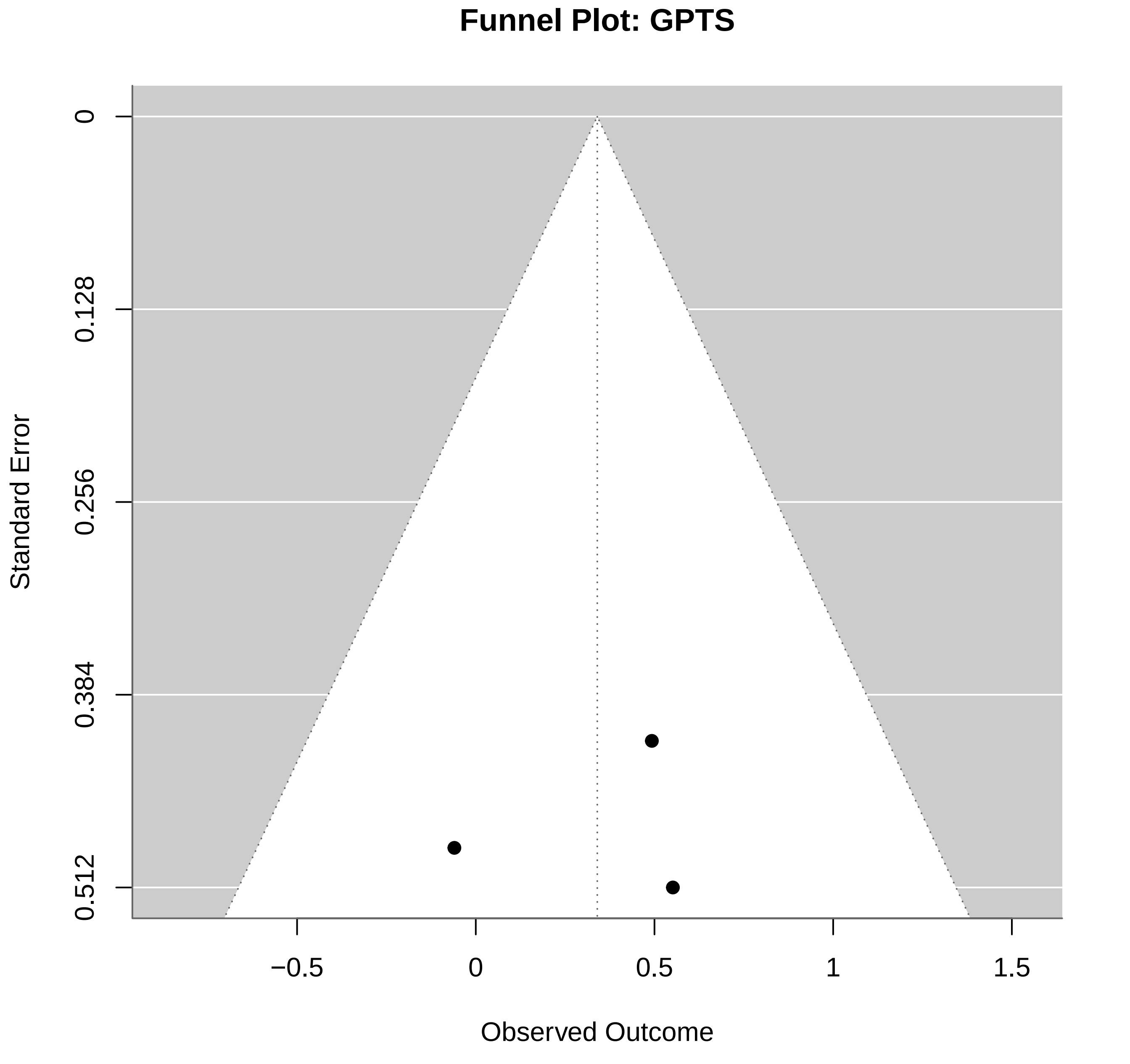

Funnel Plot: GPTS
Standard Error
0
0.128
0.256
0.384
0.512
−0.5
0
0.5
1
1.5
Observed Outcome

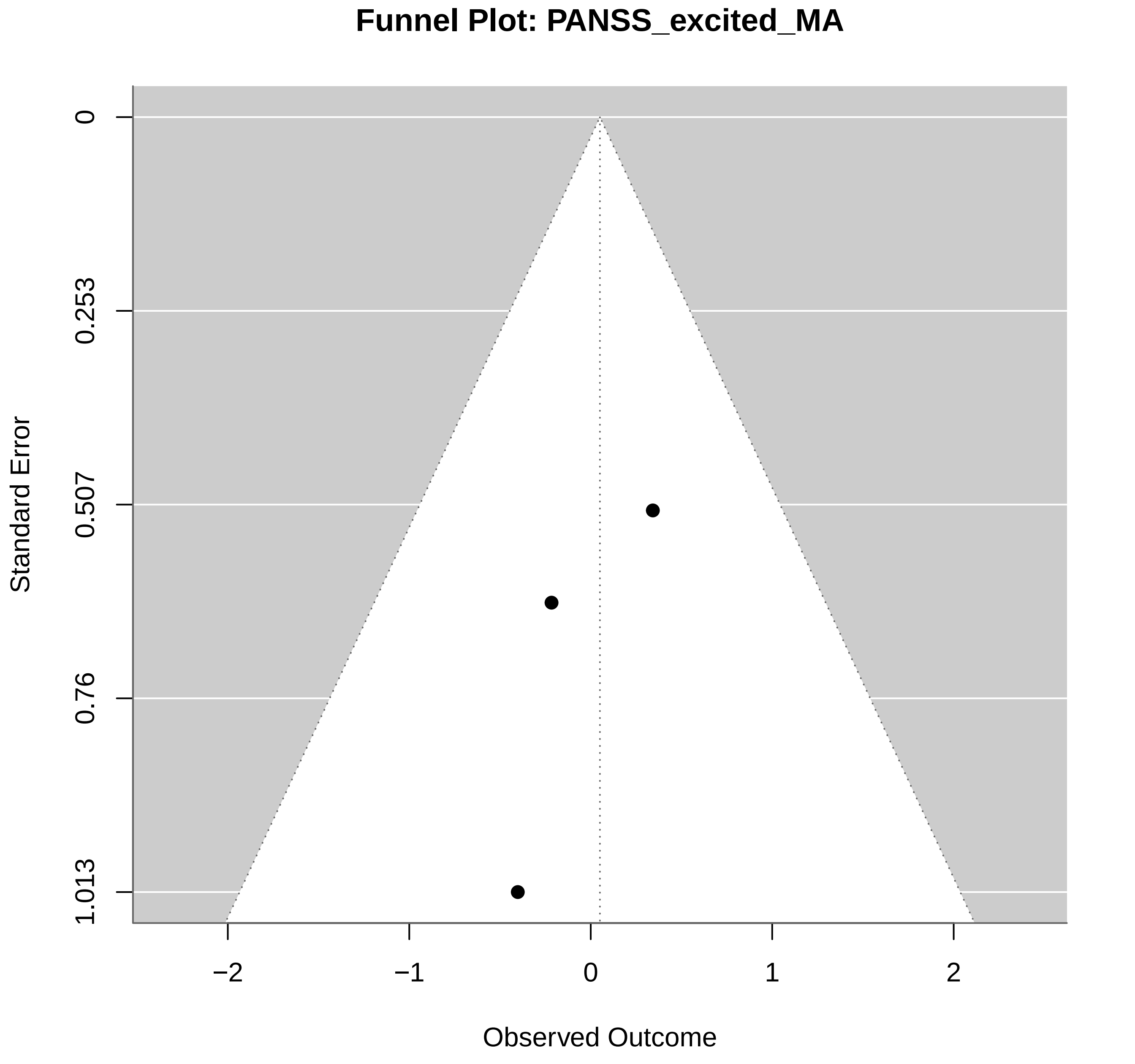

Funnel Plot: PANSS_excited_MA
Standard Error
0
0.253
0.507
0.76
1.013
−2
−1
0
1
2
Observed Outcome
