# Supplementary material for: Do virtual reality-based therapies affect symptomatology and psychosocial functioning in schizophrenia spectrum disorders: systematic review and meta-analysis
Source: BJPsych Open. 2026 Jun 18;12(4):e165. doi: 10.1192/bjo.2026.12012 (PMC13276771; doi:10.1192/bjo.2026.12012)
Supplement: Colgan et al. supplementary material 4 — Colgan et al. supplementary material [file S2056472426120122sup004.pdf]

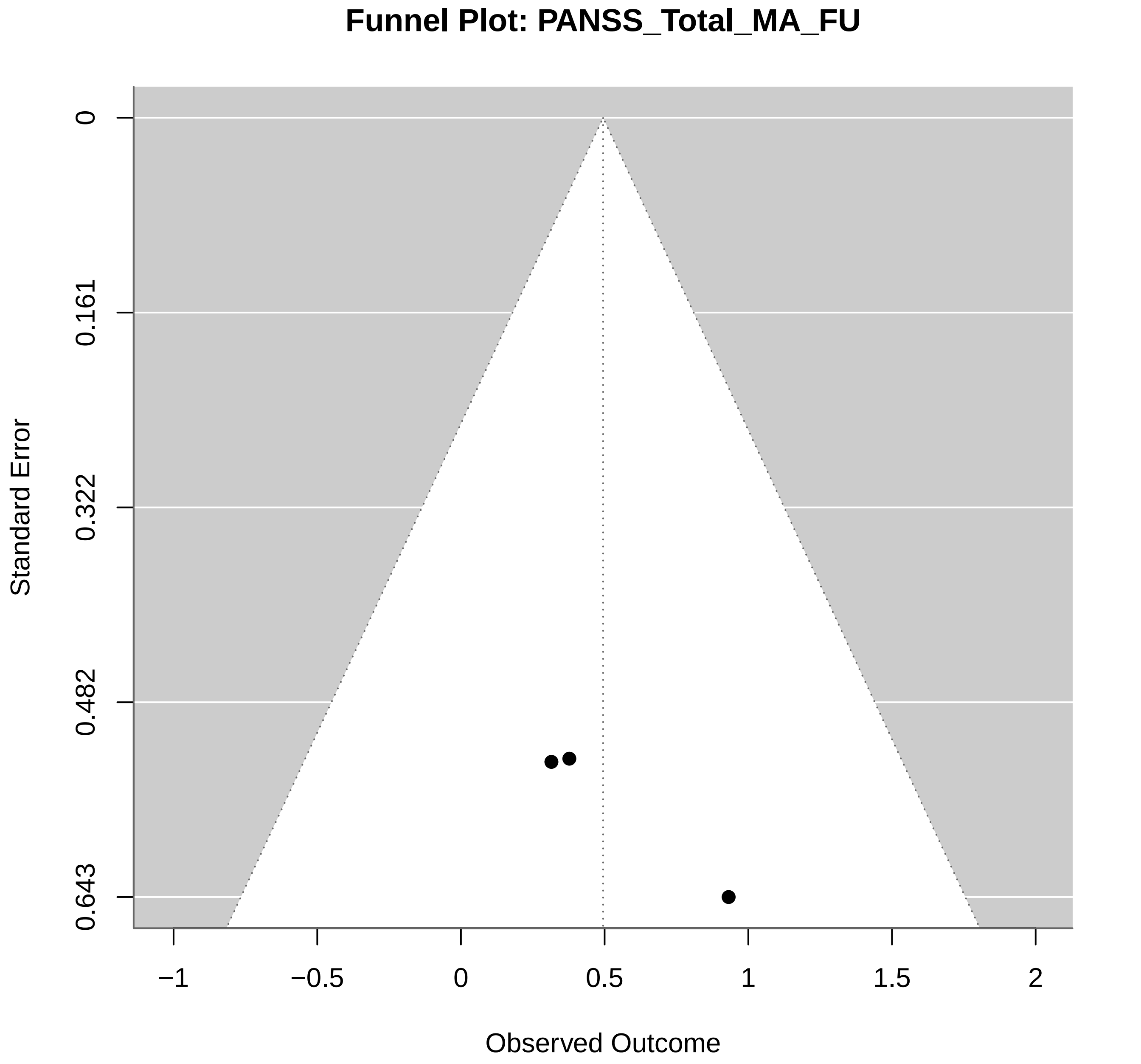

Funnel Plot: PANSS_Total_MA_FU
Standard Error
0
0.161
0.322
0.482
0.643
−1
−0.5
0
0.5
1
1.5
2
Observed Outcome

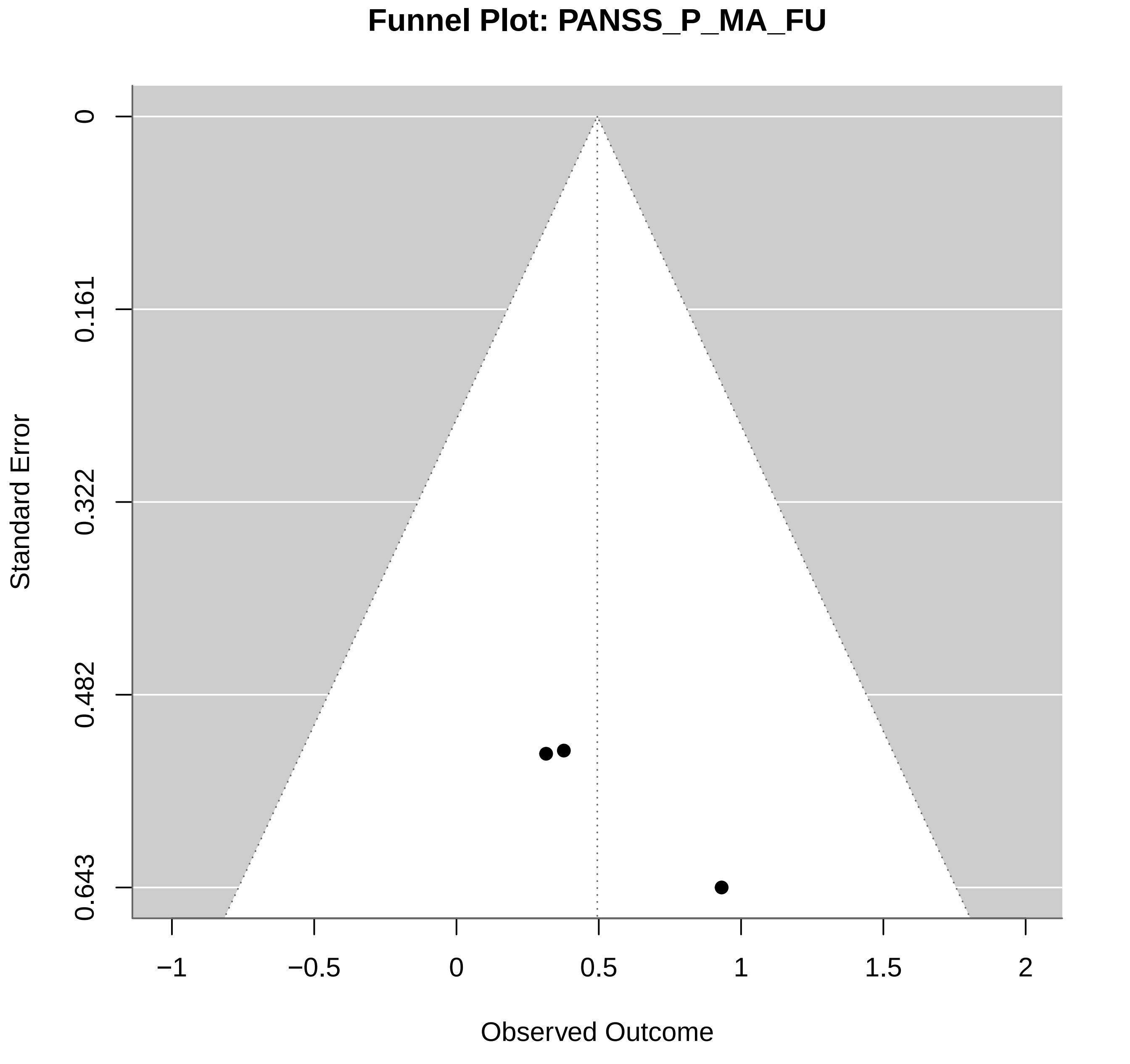

Funnel Plot: PANSS_P_MA_FU
Standard Error
0
0.161
0.322
0.482
0.643
−1
−0.5
0
0.5
1
1.5
2
Observed Outcome

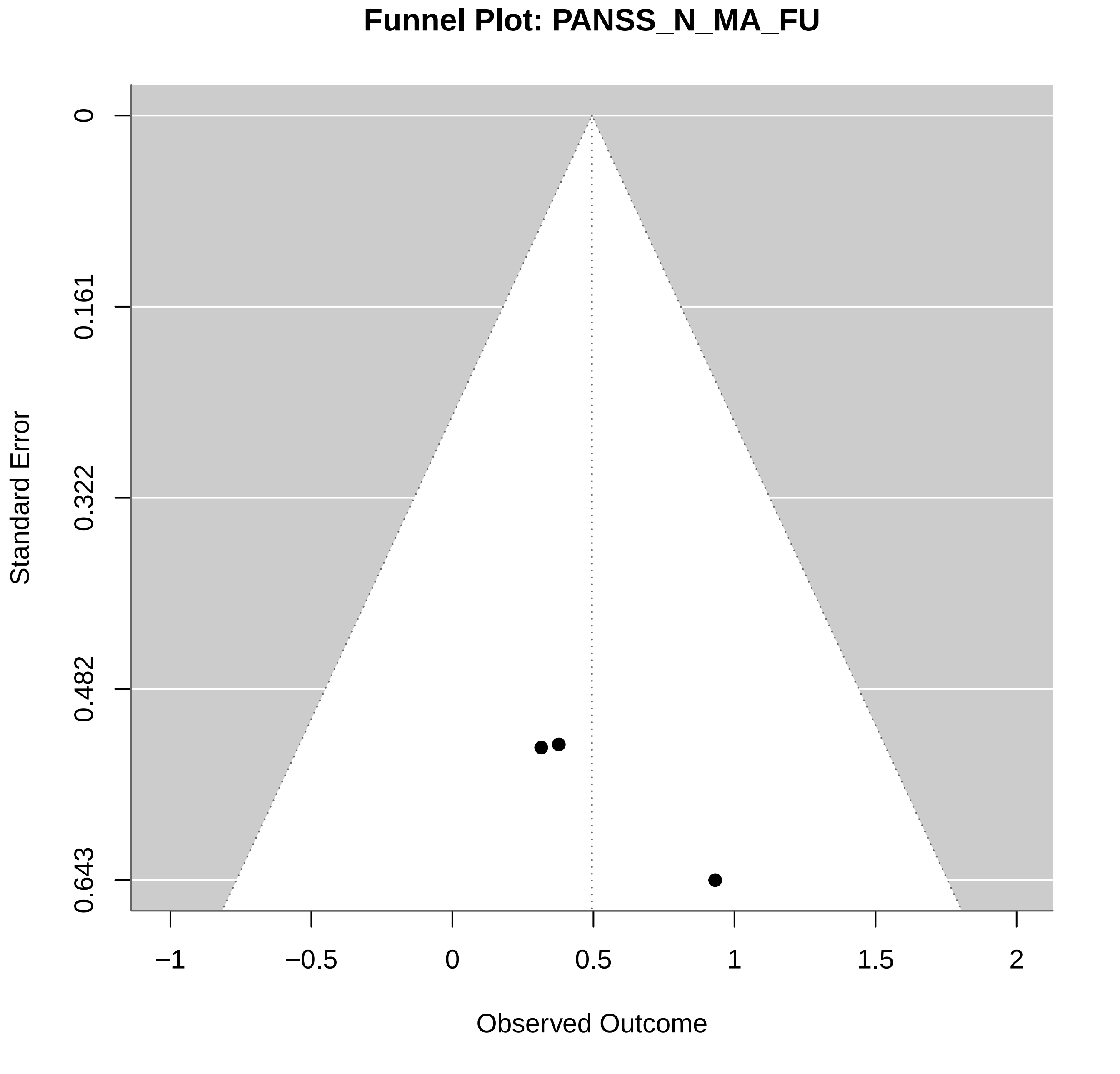
Funnel Plot: PANSS_N_MA_FU
Standard Error
0
0.161
0.322
0.482
0.643
−1
−0.5
0
0.5
1
1.5
2
Observed Outcome

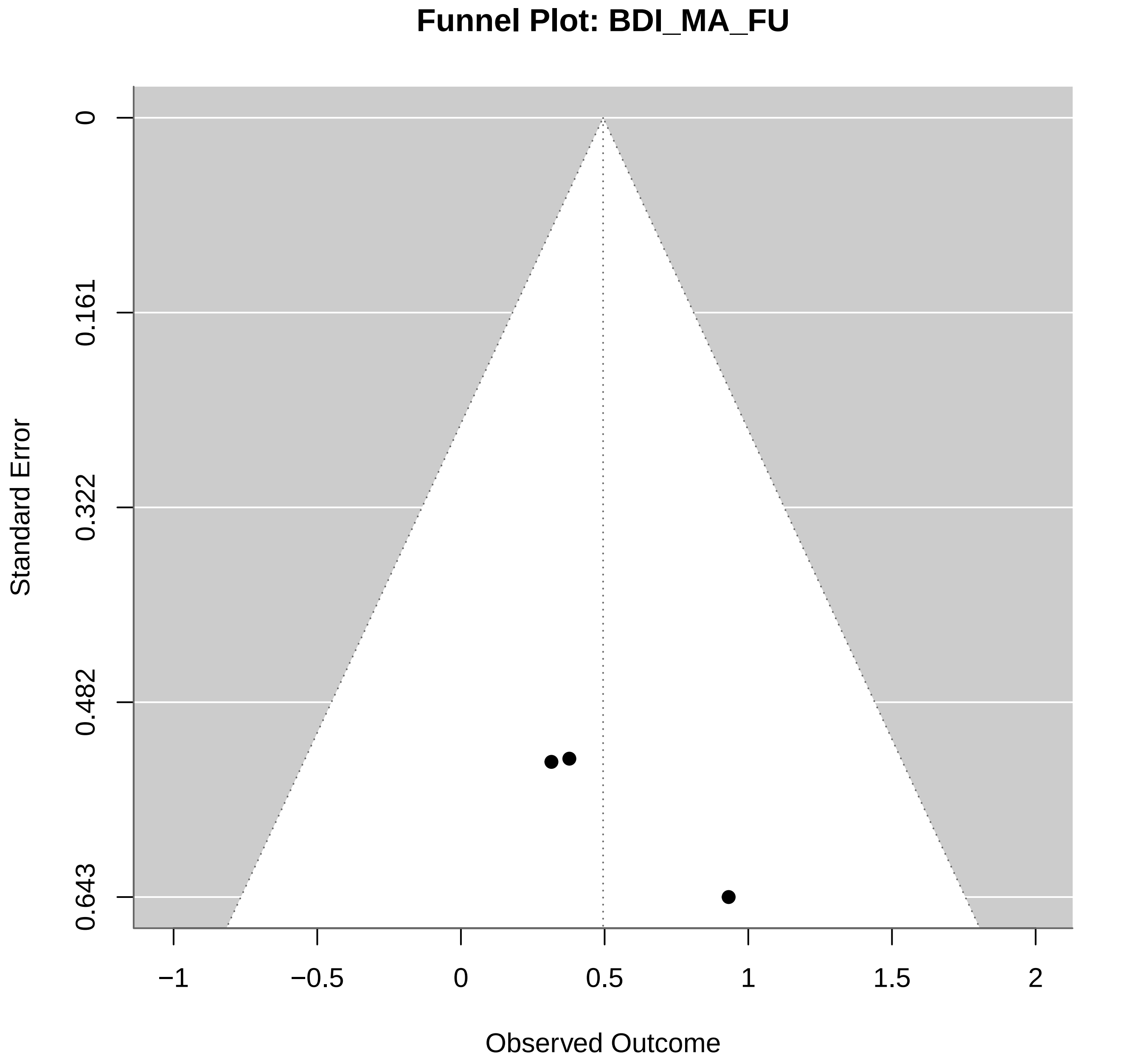
Funnel Plot: BDI_MA_FU
Standard Error
0
0.161
0.322
0.482
0.643
−1
−0.5
0
0.5
1
1.5
2
Observed Outcome
