# Supplementary material for: Do virtual reality-based therapies affect symptomatology and psychosocial functioning in schizophrenia spectrum disorders: systematic review and meta-analysis
Source: BJPsych Open. 2026 Jun 18;12(4):e165. doi: 10.1192/bjo.2026.12012 (PMC13276771; doi:10.1192/bjo.2026.12012)
Supplement: Colgan et al. supplementary material 5 — Colgan et al. supplementary material [file S2056472426120122sup005.pdf]

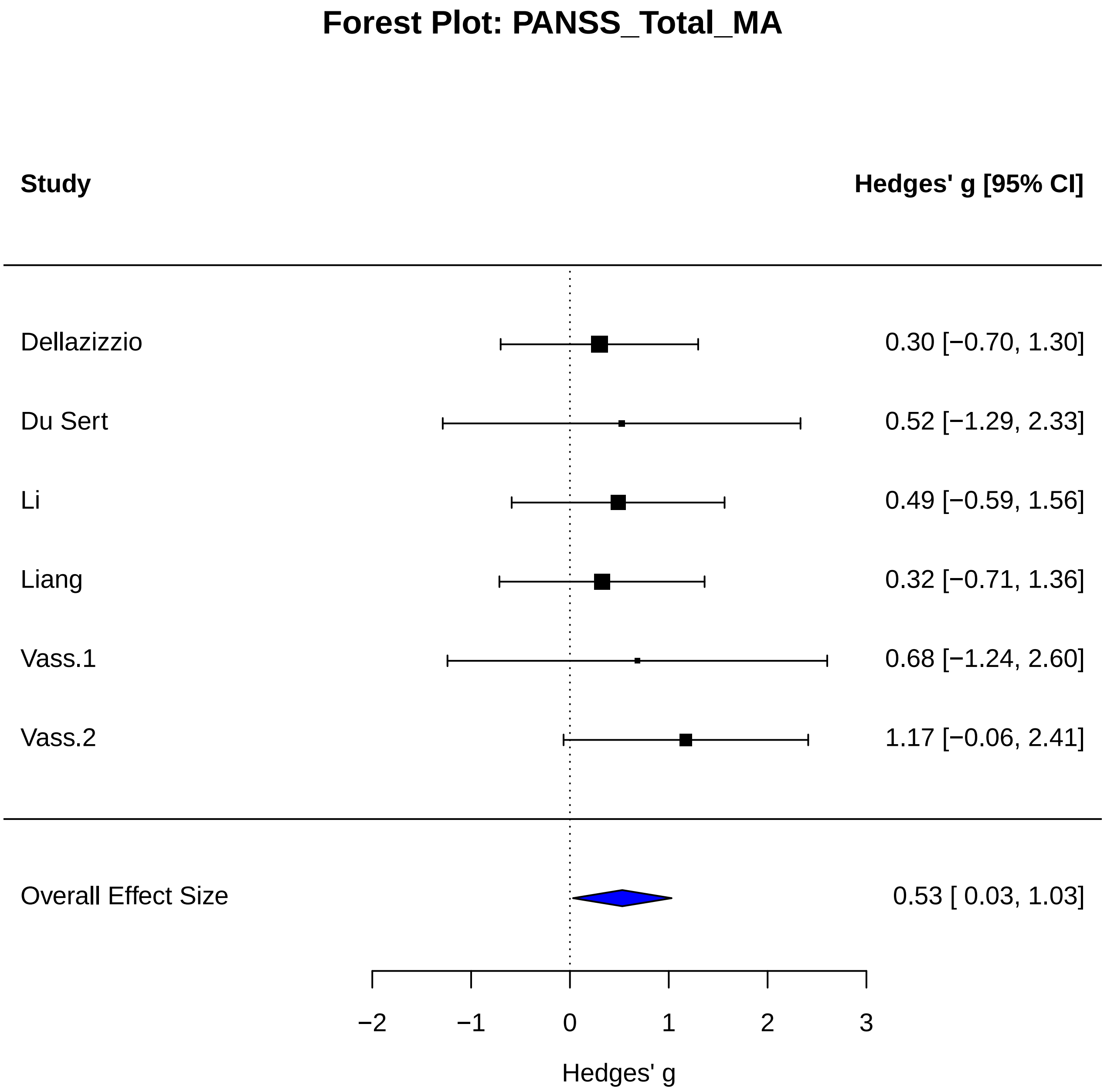
Forest Plot: PANSS_Total_MA
Study
Hedges' g [95% CI]
Dellazizzio
0.30 [−0.70, 1.30]
Du Sert
0.52 [−1.29, 2.33]
Li
0.49 [−0.59, 1.56]
Liang
0.32 [−0.71, 1.36]
Vass.1
0.68 [−1.24, 2.60]
Vass.2
1.17 [−0.06, 2.41]
Overall Effect Size
0.53 [0.03, 1.03]
−2
−1
0
1
2
3
Hedges' g

Forest Plot: PANSS_Positive_MA

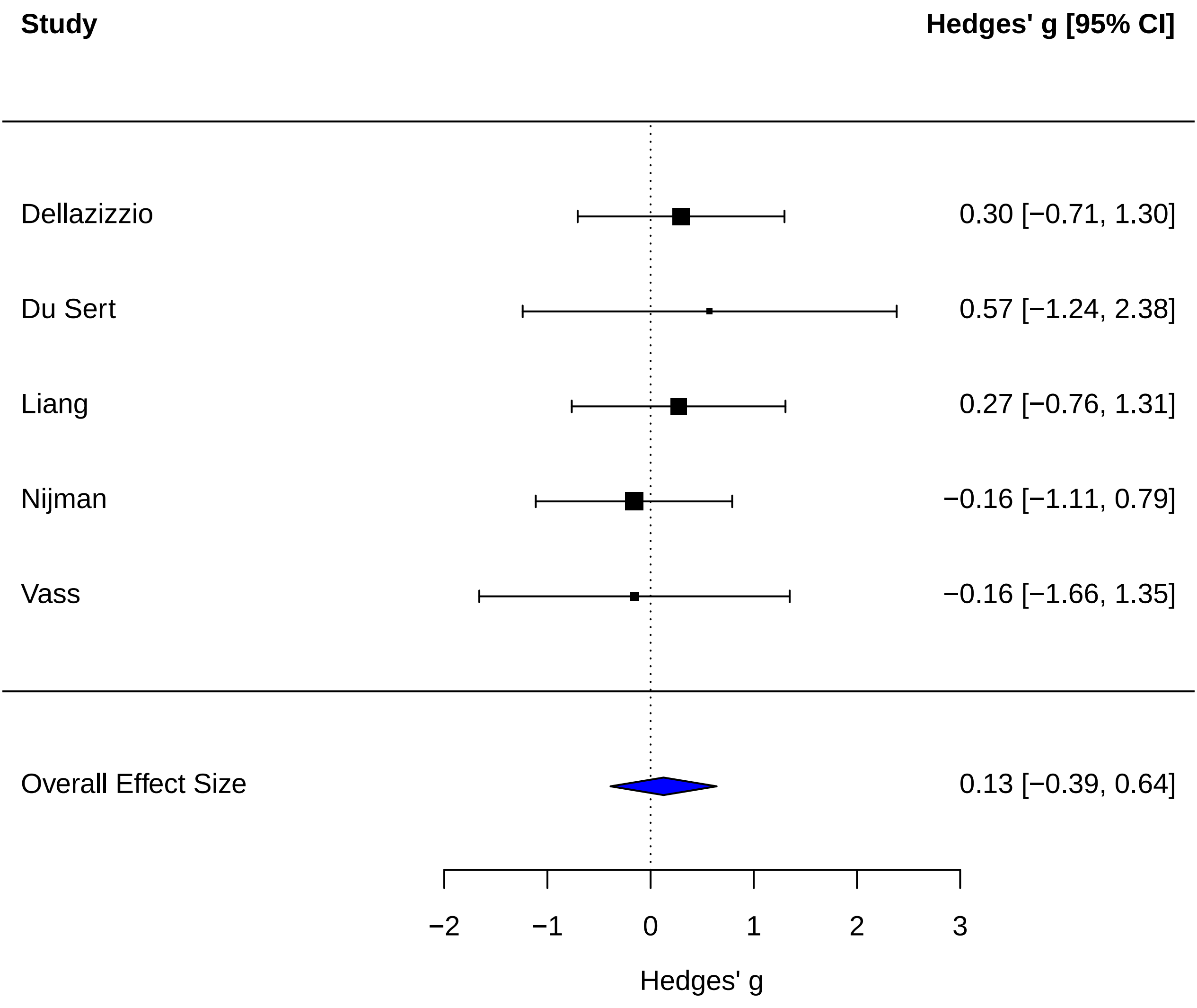

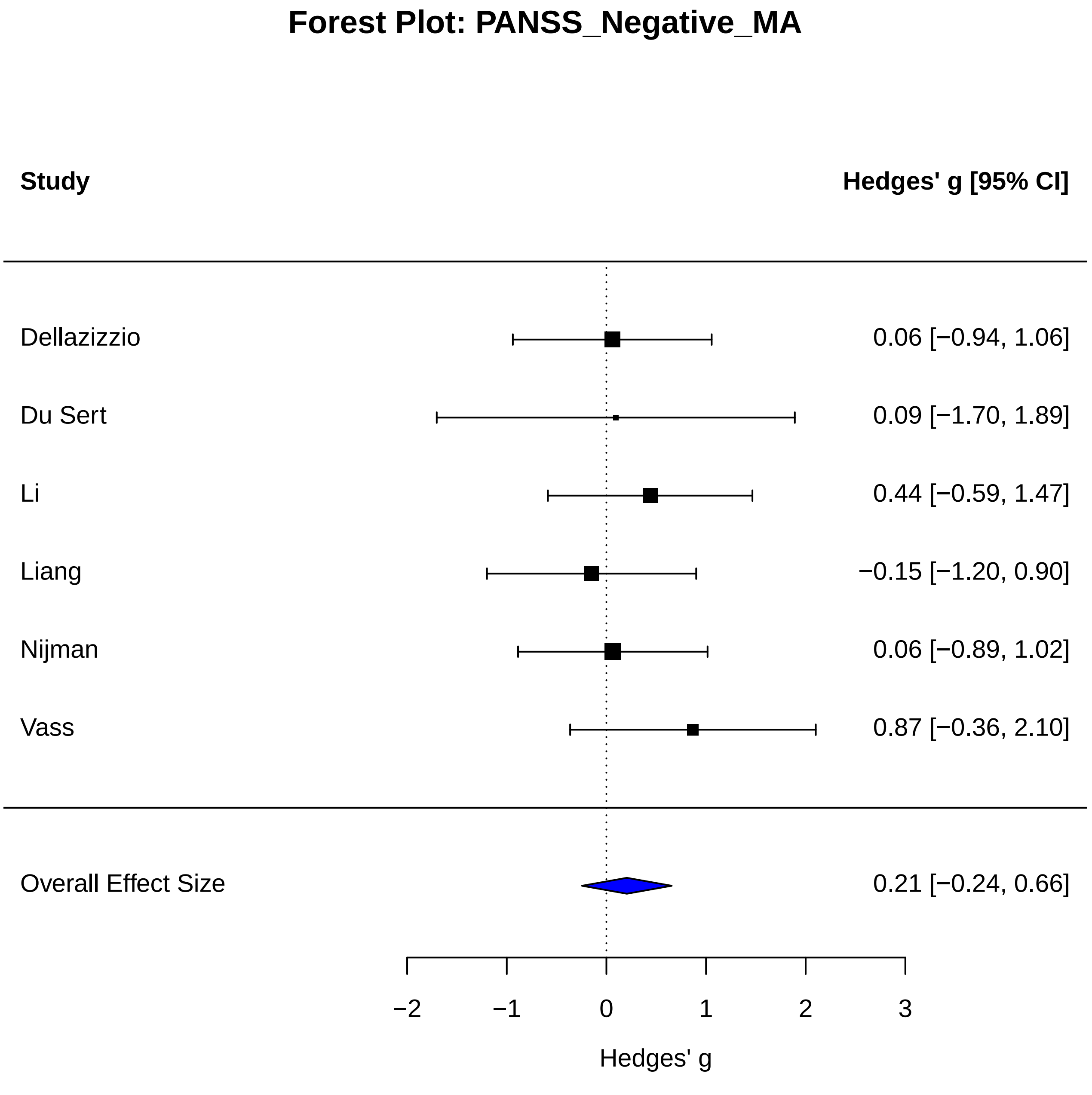
Forest Plot: PANSS_Negative_MA
Study
Hedges' g [95% CI]
Dellazizzio
0.06 [−0.94, 1.06]
Du Sert
0.09 [−1.70, 1.89]
Li
0.44 [−0.59, 1.47]
Liang
−0.15 [−1.20, 0.90]
Nijman
0.06 [−0.89, 1.02]
Vass
0.87 [−0.36, 2.10]
Overall Effect Size
0.21 [−0.24, 0.66]
−2
−1
0
1
2
3
Hedges' g

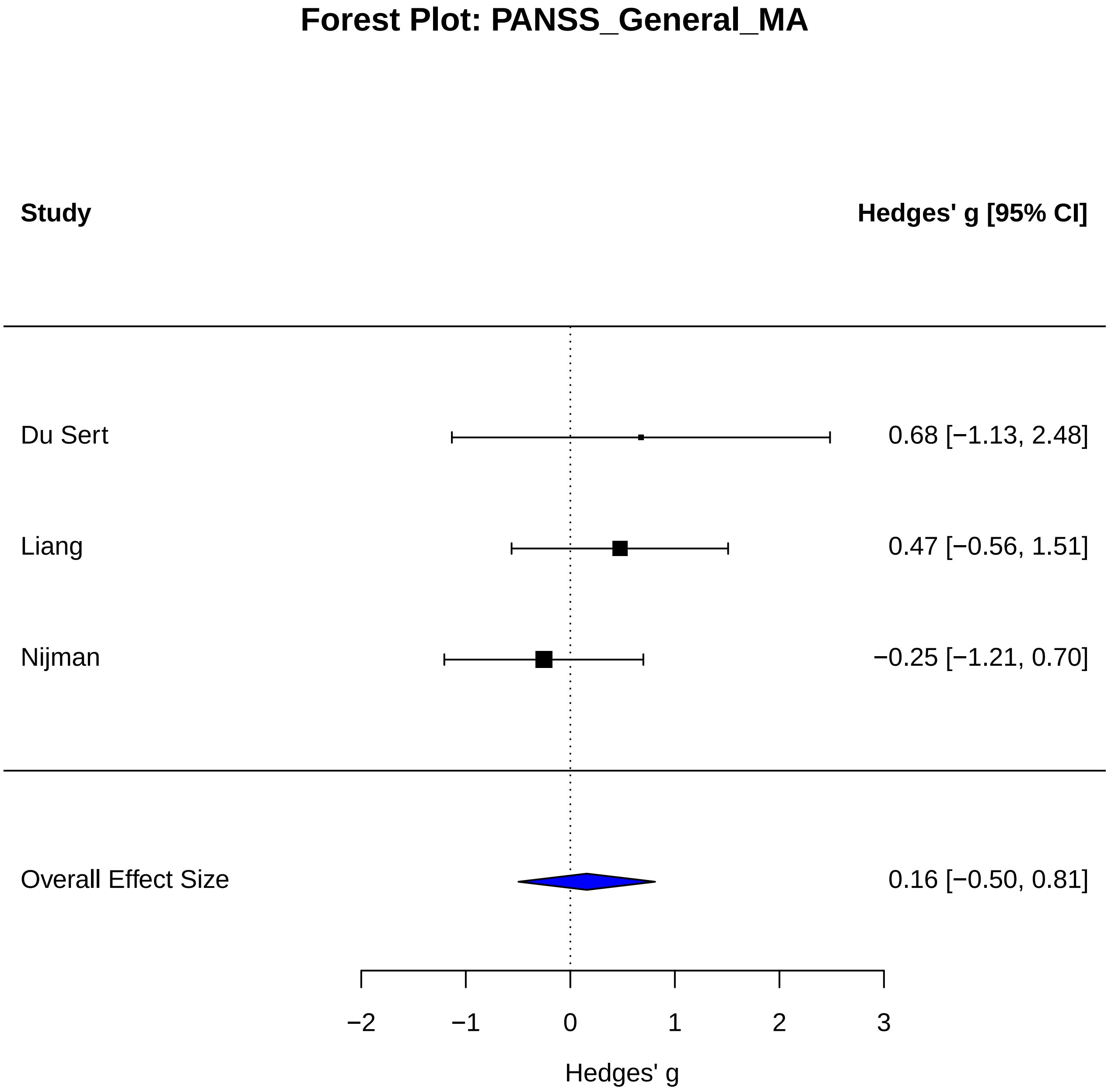
Forest Plot: PANSS_General_MA
Study
Hedges' g [95% CI]
Du Sert
0.68 [−1.13, 2.48]
Liang
0.47 [−0.56, 1.51]
Nijman
−0.25 [−1.21, 0.70]
Overall Effect Size
0.16 [−0.50, 0.81]
−2
−1
0
1
2
3
Hedges' g

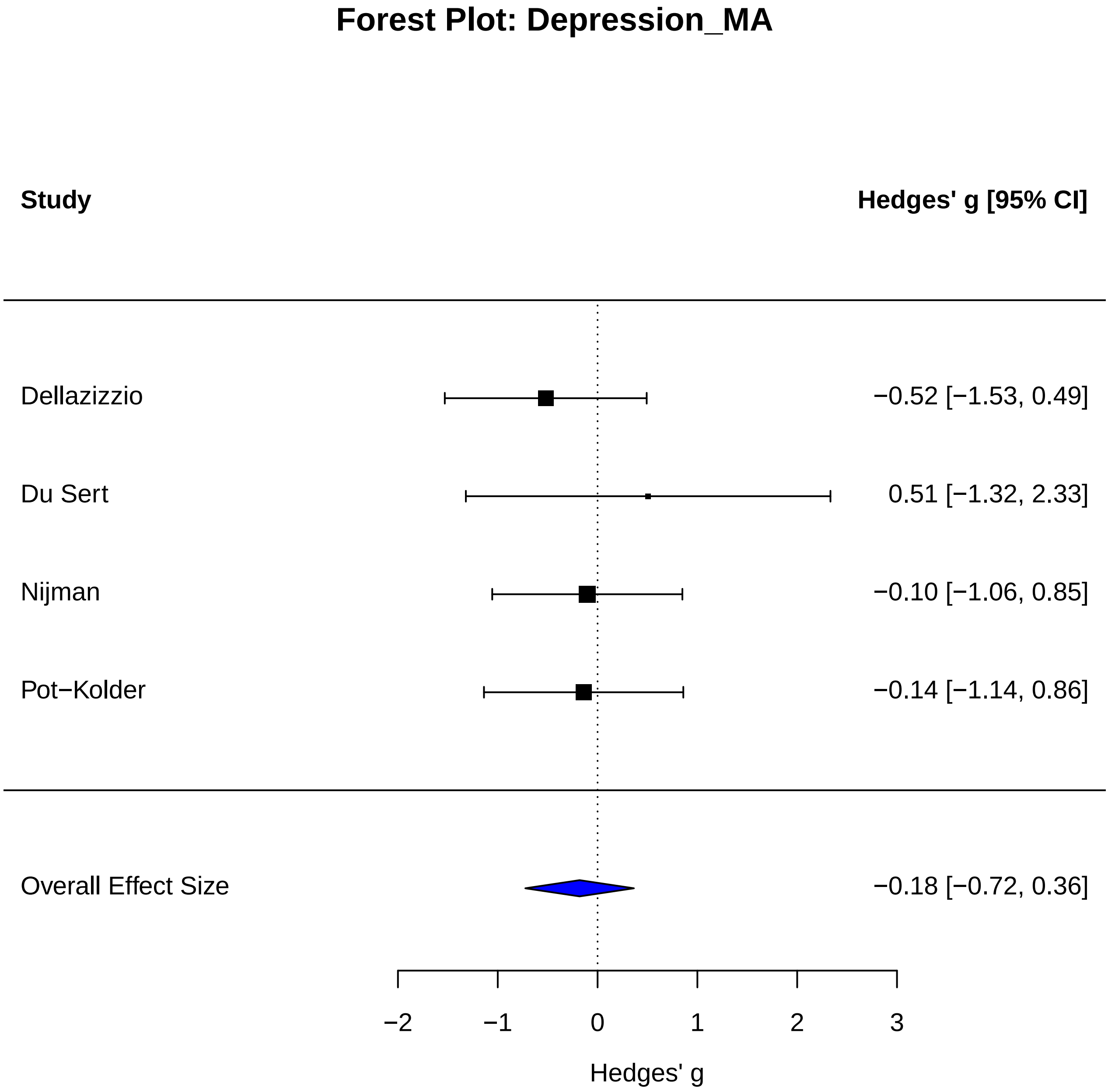
Forest Plot: Depression_MA
Study
Hedges' g [95% CI]
Dellazizzio
−0.52 [−1.53, 0.49]
Du Sert
0.51 [−1.32, 2.33]
Nijman
−0.10 [−1.06, 0.85]
Pot−Kolder
−0.14 [−1.14, 0.86]
Overall Effect Size
−0.18 [−0.72, 0.36]
−2
−1
0
1
2
3
Hedges' g

Forest Plot: GPTS

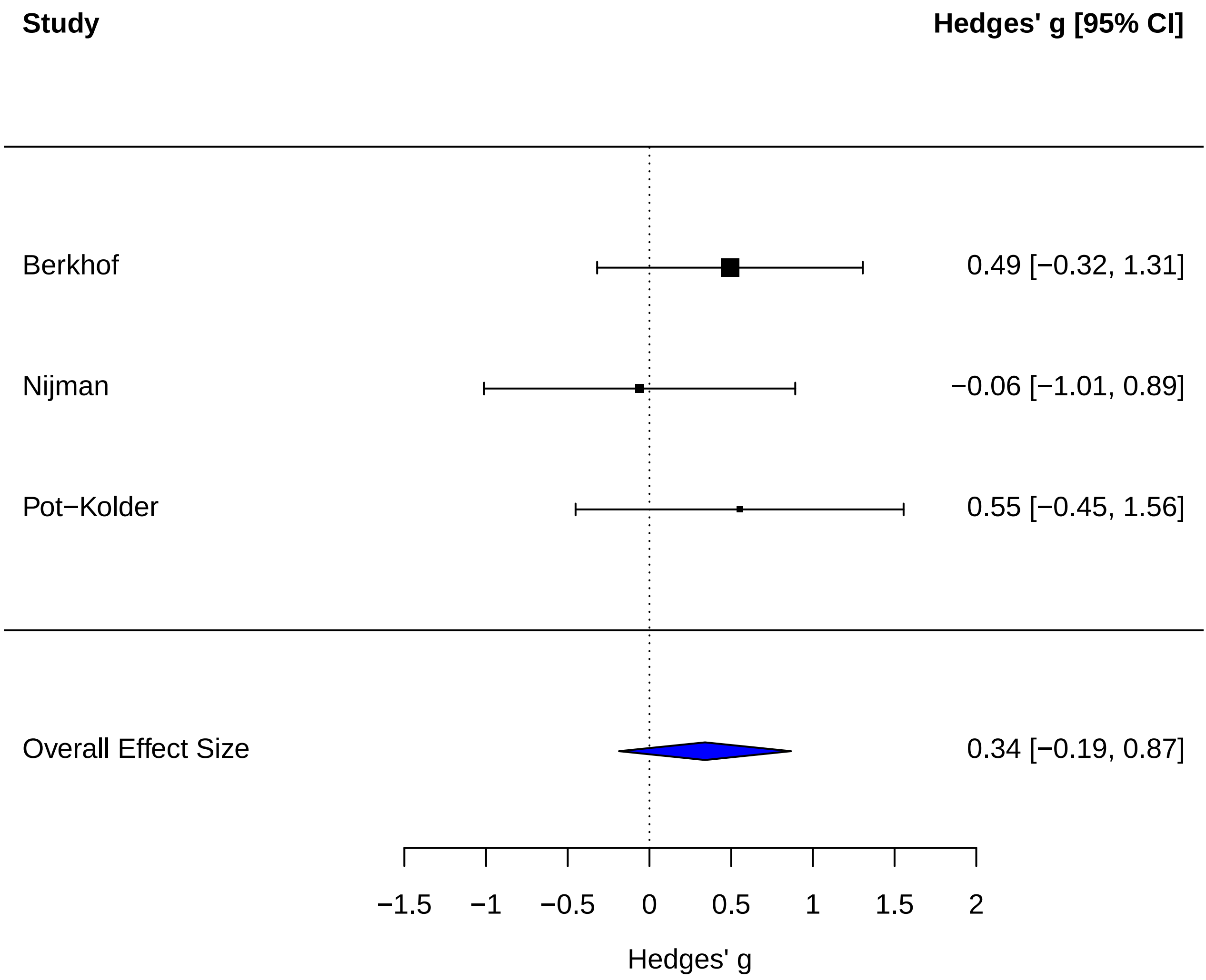

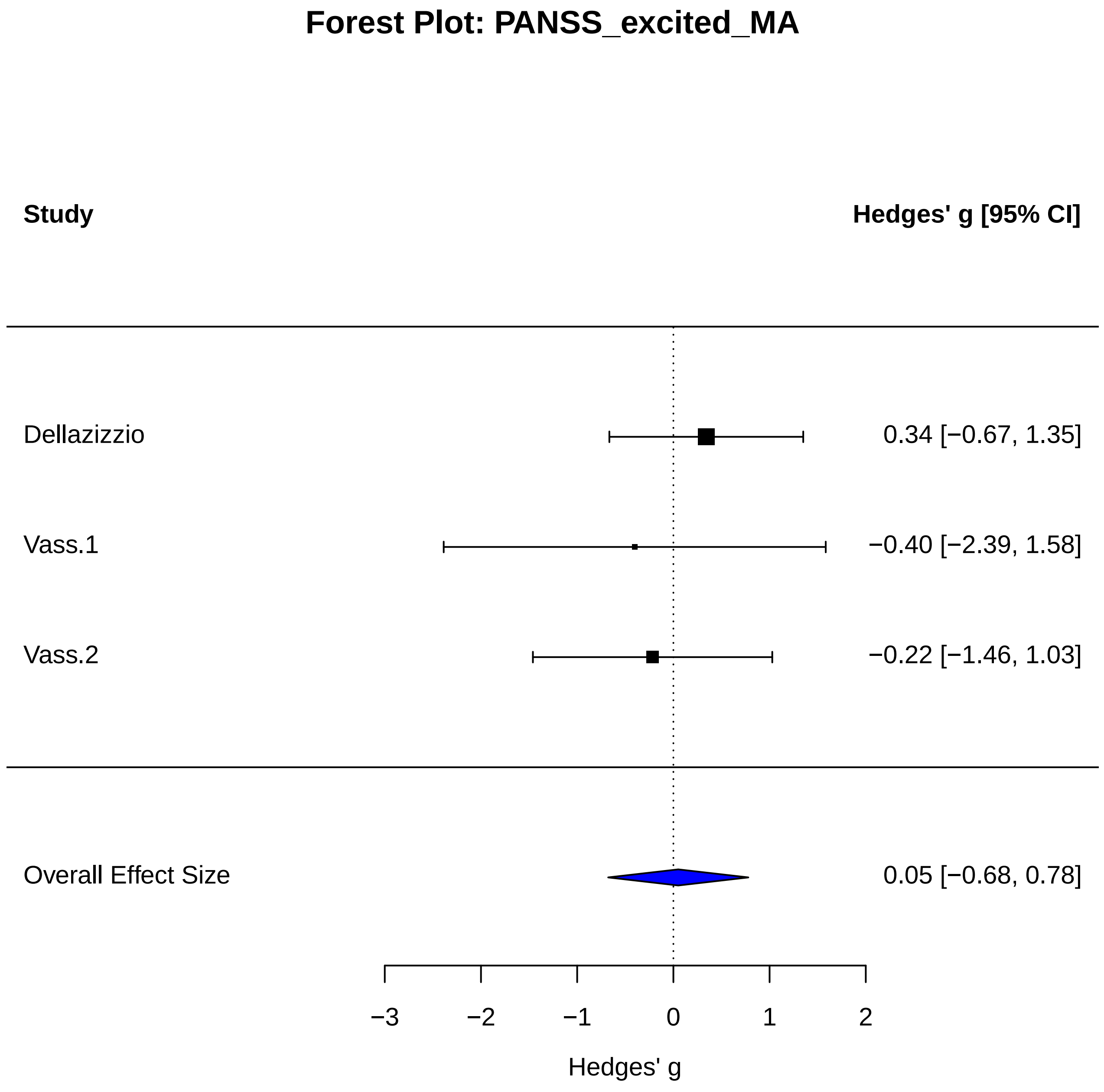
Forest Plot: PANSS_excited_MA
Study
Hedges' g [95% CI]
Dellazizzio
0.34 [−0.67, 1.35]
Vass.1
−0.40 [−2.39, 1.58]
Vass.2
−0.22 [−1.46, 1.03]
Overall Effect Size
0.05 [−0.68, 0.78]
−3
−2
−1
0
1
2
Hedges' g
