# Supplementary material for: Do virtual reality-based therapies affect symptomatology and psychosocial functioning in schizophrenia spectrum disorders: systematic review and meta-analysis
Source: BJPsych Open. 2026 Jun 18;12(4):e165. doi: 10.1192/bjo.2026.12012 (PMC13276771; doi:10.1192/bjo.2026.12012)

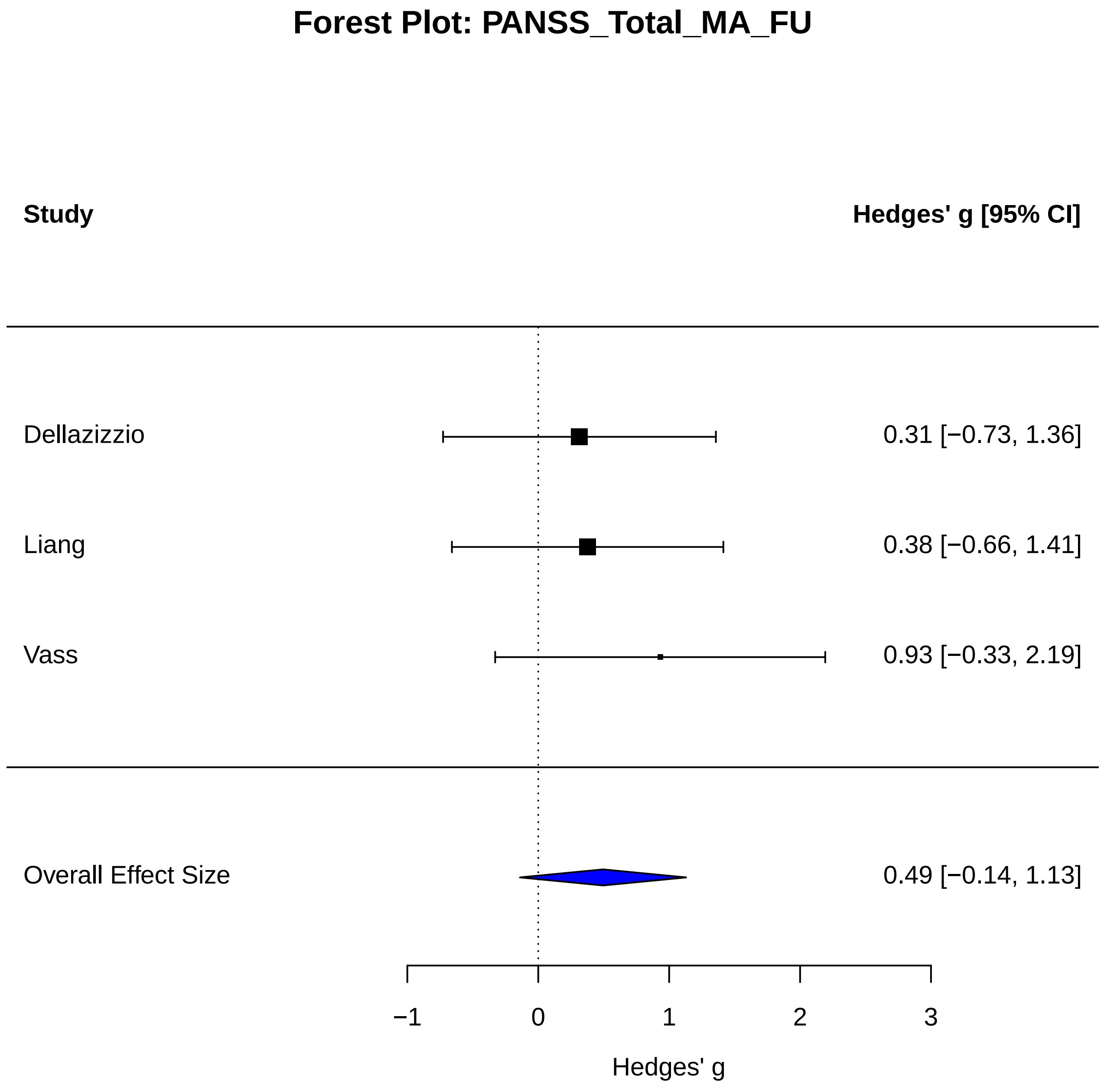
Forest Plot: PANSS_Total_MA_FU
Study
Hedges' g [95% CI]
Dellazizzio
0.31 [−0.73, 1.36]
Liang
0.38 [−0.66, 1.41]
Vass
0.93 [−0.33, 2.19]
Overall Effect Size
0.49 [−0.14, 1.13]
−1
0
1
2
3
Hedges' g

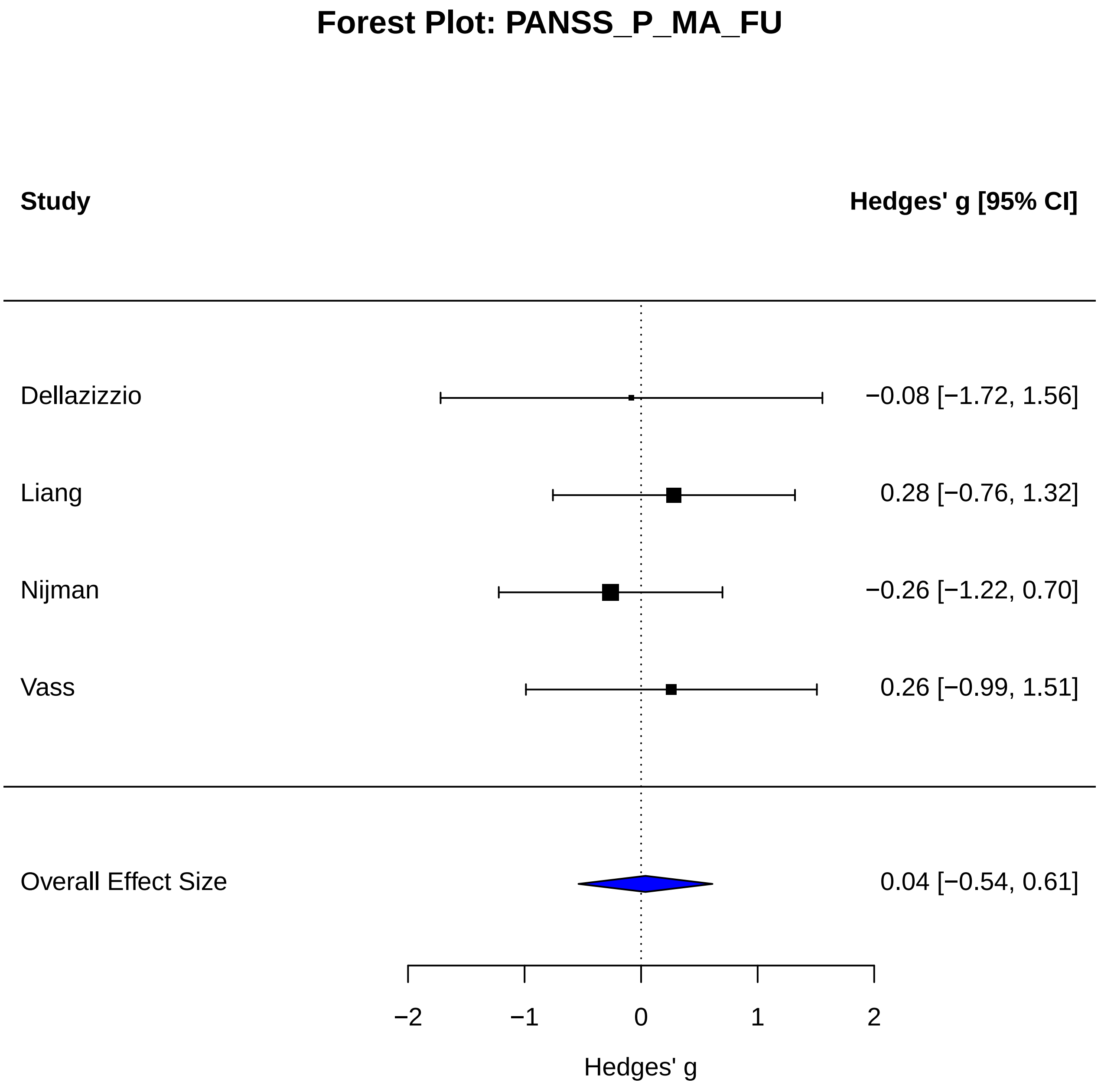
Forest Plot: PANSS_P_MA_FU
Study
Hedges' g [95% CI]
Dellazizzio
−0.08 [−1.72, 1.56]
Liang
0.28 [−0.76, 1.32]
Nijman
−0.26 [−1.22, 0.70]
Vass
0.26 [−0.99, 1.51]
Overall Effect Size
0.04 [−0.54, 0.61]
−2
−1
0
1
2
Hedges' g

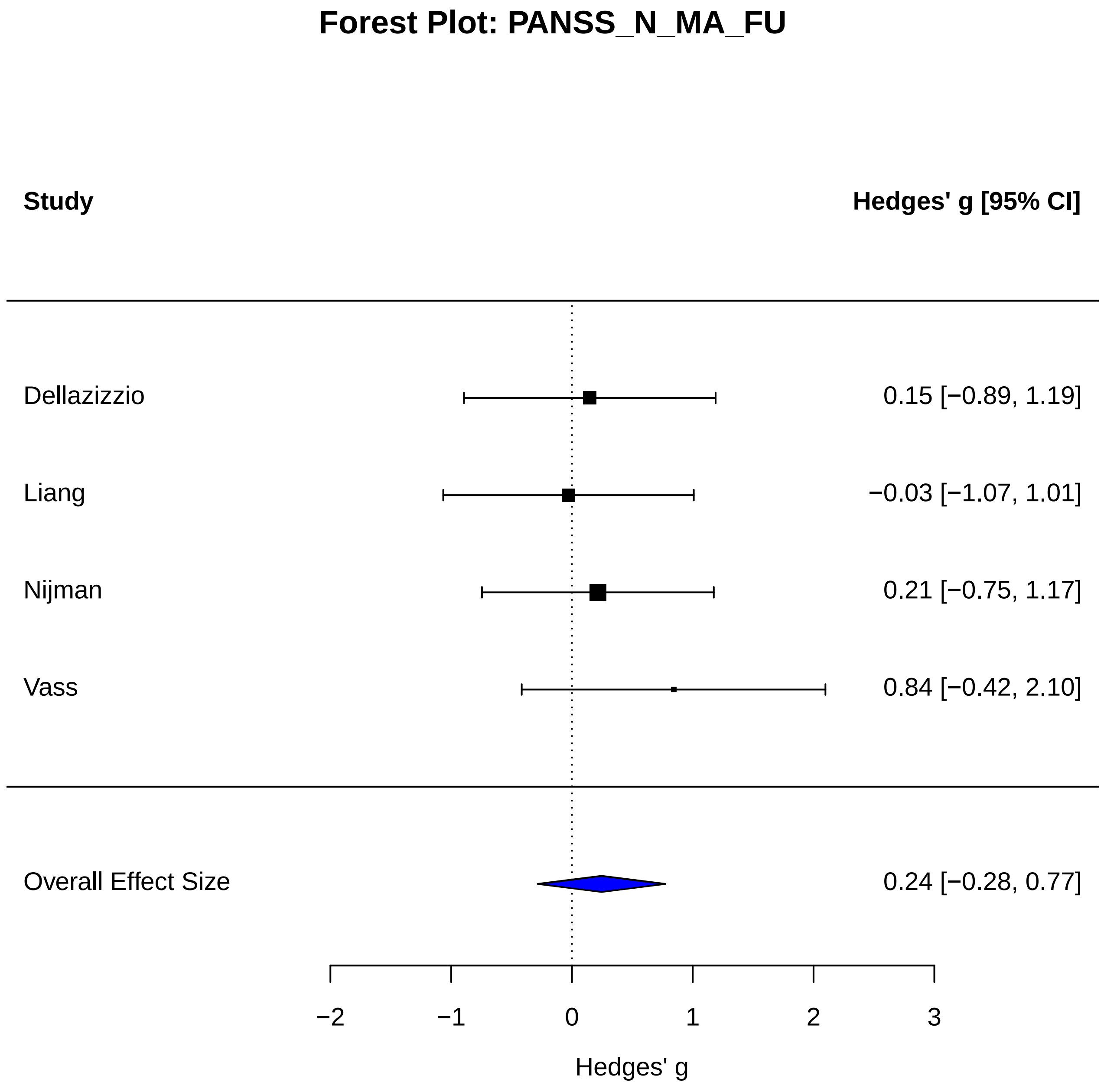
Forest Plot: PANSS_N_MA_FU
Study
Hedges' g [95% CI]
Dellazizzio
0.15 [−0.89, 1.19]
Liang
−0.03 [−1.07, 1.01]
Nijman
0.21 [−0.75, 1.17]
Vass
0.84 [−0.42, 2.10]
Overall Effect Size
0.24 [−0.28, 0.77]
−2
−1
0
1
2
3
Hedges' g

Forest Plot: BDI_MA_FU

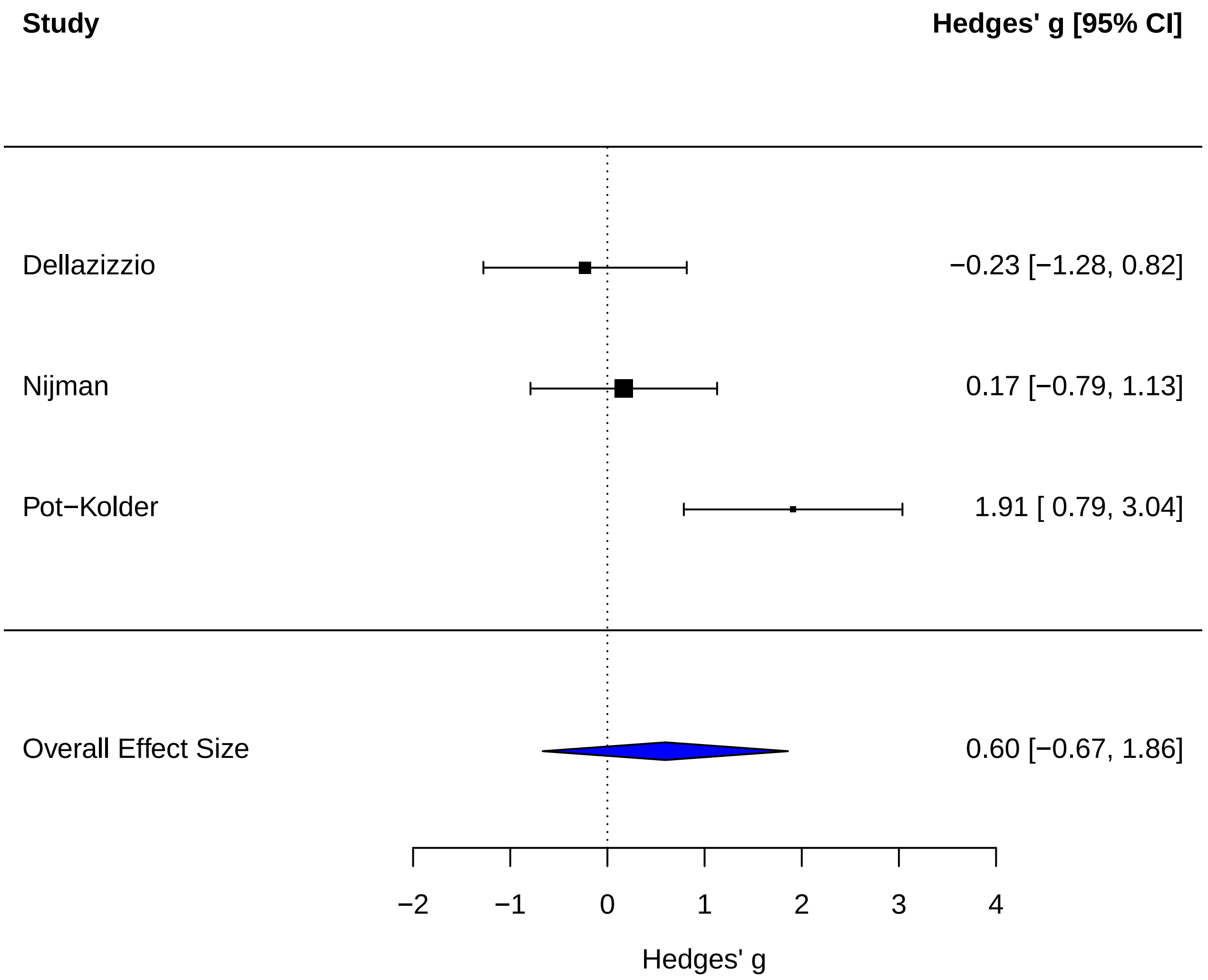

Supplement: Colgan et al. supplementary material 6 — Colgan et al. supplementary material [file S2056472426120122sup006.pdf]
