# Supplementary material for: Do virtual reality-based therapies affect symptomatology and psychosocial functioning in schizophrenia spectrum disorders: systematic review and meta-analysis
Source: BJPsych Open. 2026 Jun 18;12(4):e165. doi: 10.1192/bjo.2026.12012 (PMC13276771; doi:10.1192/bjo.2026.12012)
Supplement: Colgan et al. supplementary material 7 — Colgan et al. supplementary material [file S2056472426120122sup007.docx]

Supplementary Table 1: Sensitivity analyses for baseline to endpoint

| Outcome | *k* studies | *n* participants  (*n* intervention; *n* control) | Original meta-analysis | | Meta-analysis with Knapp–Hartung adjustment | |
| --- | --- | --- | --- | --- | --- | --- |
|  |  |  | Hedge’s g  (95% CI) | p-value | Hedge’s g  (95% CI) | p-value |
| Overall psychosis symptoms | 6 | 263  (134; 129) | 0.53  (0.03; 1.03) | 0.037 | 0.53  (0.18-0.88) | 0.011 |
| Positive psychosis symptoms | 6 | 314  (159; 155) | 0.13  (-0.39; 0.64) | 0.632 | 0.13  (-0.22-0.47) | 0.371 |
| Negative psychosis symptoms | 6 | 314  (159; 155) | 0.21  (-0.25; 0.66) | 0.372 | 0.21  (-0.16-0.57) | 0.21 |
| General psychopathy | 3 | 210  (109; 101) | 0.16  (-0.50; 0.81) | 0.637 | 0.16  (-1.04-1.35) | 0.629 |
| Depression | 4 | 214  (107; 107) | -0.18  (-0.72; 0.36) | 0.516 | -0.18  (-0.69-0.33) | 0.344 |
| GPTS A | 3 | 225  (105; 120) | 0.34  (-0.19; 0.87) | 0.206 | 0.34  (-0.47-1.15) | 0.213 |
| Excited psychosis symptoms | 3 | 121  (59; 62) | 0.05  (-0.68; 0.78) | 0.892 | 0.05  (-0.89-1) | 0.839 |

Supplementary Table 2: Sensitivity analyses for baseline to 12 month follow up

| Outcome | *k* studies | *n* participants  (*n* intervention; *n* control) | Original meta-analysis | | Meta-analysis with Knapp–Hartung adjustment | |
| --- | --- | --- | --- | --- | --- | --- |
|  |  |  | Hedge’s g  (95% CI) | p-value | Hedge’s g  (95% CI) | p-value |
| Overall psychosis symptoms | 3 | 155  (78; 77) | 0.50  (-0.14; 1.13) | 0.127 | 0.50  (-0.28-1.27) | 0.112 |
| Positive psychosis symptoms | 4 | 222  (111; 111) | 0.04  (-0.54; 0.61) | 0.899 | 0.04 (-0.42-0.5) | 0.812 |
| Negative psychosis symptoms | 4 | 222  (111; 111) | 0.25  (-0.28; 0.77) | 0.364 | 0.25  (-0.29-0.78) | 0.242 |
| Depression | 3 | 181  (87; 94) | 0.60  (-0.67; 1.86) | 0.354 | 0.6  (-2.2-3.4) | 0.456 |
